# Genotyping-Guided Discovery of Persiamycin A From Sponge-Associated Halophilic *Streptomonospora* sp. PA3

**DOI:** 10.3389/fmicb.2020.01237

**Published:** 2020-06-09

**Authors:** Soheila Matroodi, Vilja Siitonen, Bikash Baral, Keith Yamada, Amir Akhgari, Mikko Metsä-Ketelä

**Affiliations:** ^1^Laboratory of Biotechnology, Department of Marine Biology, Faculty of Marine Science and Oceanography, Khorramshahr University of Marine Science and Technology, Khorramshahr, Iran; ^2^Laboratory of Antibiotic Biosynthesis Engineering, Department of Biochemistry, University of Turku, Turku, Finland

**Keywords:** actinomycetes, aromatic polyketides, marine sponges, Persian Gulf, *Streptomonospora*

## Abstract

Microbial natural products have been a cornerstone of the pharmaceutical industry, but the supply of novel bioactive secondary metabolites has diminished due to extensive exploration of the most easily accessible sources, namely terrestrial *Streptomyces* species. The Persian Gulf is a unique habitat for marine sponges, which contain diverse communities of microorganisms including marine Actinobacteria. These exotic ecosystems may cradle rare actinomycetes with high potential to produce novel secondary metabolites. In this study, we harvested 12 different species of sponges from two locations in the Persian Gulf and isolated 45 symbiotic actinomycetes to assess their biodiversity and sponge-microbe relationships. The isolates were classified into *Nocardiopsis* (24 isolates), *Streptomyces* (17 isolates) and rare genera (4 isolates) by 16S rRNA sequencing. Antibiotic activity tests revealed that culture extracts from half of the isolates displayed growth inhibitory effects against seven pathogenic bacteria. Next, we identified five strains with the genetic potential to produce aromatic polyketides by genotyping ketosynthase genes responsible for synthesis of carbon scaffolds. The combined data led us to focus on *Streptomonospora* sp. PA3, since the genus has rarely been examined for its capacity to produce secondary metabolites. Analysis of culture extracts led to the discovery of a new bioactive aromatic polyketide denoted persiamycin A and 1-hydroxy-4-methoxy-2-naphthoic acid. The genome harbored seven gene clusters involved in secondary metabolism, including a tetracenomycin-type polyketide synthase pathway likely involved in persiamycin formation. The work demonstrates the use of multivariate data and underexplored ecological niches to guide the drug discovery process for antibiotics and anticancer agents.

## Introduction

Microbial natural products have been instrumental in the development of modern medicine (Newman and Cragg, 2016). Approximately two-thirds of antibiotics and one-third of antiproliferative agents in clinical use are microbial natural products or their semi-synthetic derivatives (Newman and Cragg, 2016). In particular, actinomycetes have been a rich source of complex chemical entities that have served successfully as drug leads (Jensen et al., 2005). However, decades of work have led to a situation where traditional screening methods (e.g., bioactivity-guided isolation) have been rendered ineffective (Pye et al., 2017) due to diminishing returns and the frequent rediscovery rate of known compounds. The issue is particularly problematic for the much-studied terrestrial *Streptomyces* bacteria that have been widely used in the pharmaceutical industry (Genilloud, 2017). One attractive means to circumvent the issue has been to focus on neglected or rare source organisms found from diverse environmental habitats (Subramani and Aalbersberg, 2013). In addition, the development of alternative strategies, such as genomics (Harvey et al., 2015) and metabolomics-guided methods (Wu et al., 2015), metagenomic approaches to access the chemistry of uncultivable organisms (Katz et al., 2016) and manipulation of natural metabolism to awaken cryptic pathways (Baral et al., 2018), have ensured the continued discovery of novel natural products. The advances in next generation genome sequencing combined with improved bioinformatic tools, e.g., antiSMASH (Blin et al., 2019) and MIBiG (Medema et al., 2015), have allowed the identification of new biosynthetic gene clusters and natural products (Naughton et al., 2017; Malmierca et al., 2018). For example, this approach, known as genome mining, has been used for the identification of new antibiotic scaffolds with a high degree of novelty and diversity from 21 rare marine actinomycetes (Schorn et al., 2016). These analyses have revealed that some bacteria possess the potential to produce more secondary metabolites than are normally found under standard laboratory conditions (Baral et al., 2018; Sekurova et al., 2019).

Over the past decade, marine organisms have become an important source of remarkably diverse and unusual, often highly complex, natural products (Carroll et al., 2019). In fact, the number of new chemical structures reported from marine organisms has continuously increased with over 200 novel metabolites described every year (Raimundo et al., 2018). Marine sponge species (phylum Porifera) host diverse microbial populations in their mesohyl matrix that include bacteria, fungi and archaea, as well as viruses (Xu X. N. et al., 2018; Achlatis et al., 2019), which may constitute 35–60% of the sponge volume. Marine sponges harbor at least 32 bacterial phyla including Acidobacteria, Actinobacteria, Cyanobacteria and Proteobacteria (Abdelmohsen et al., 2014). Recent data has proposed that the symbionts play crucial roles in sponge chemistry and production of pharmaceutically relevant bioactive metabolites (Thomas et al., 2010).

Marine actinomycete species have adapted specifically to the oceanic environment (e.g., to high salinity and pressure) (Hamedi et al., 2013; Zhang et al., 2015). It has been shown that these species are physiologically and phylogenetically distinct from their terrestrial relatives, and they represent a rich source for novel, chemically diverse bioactive secondary metabolites with potential applications in antimicrobial and anticancer therapy (Bull and Stach, 2007; Xu D. et al., 2018). The discovery of new natural products from marine actinomycetes has increased more than from terrestrial actinomycetes over the past two decades (Subramani and Aalbersberg, 2013). Marine sponges in particular have become a notable environment for finding rare and new actinomycete genera including species from *Actinomadura*, *Amycolatopsis, Pseudonocardia*, *Saccharomonospora, Saccharopolyspora* and *Streptomonospora* (Passari et al., 2018).

The Persian Gulf, which is bordered by the Arabian Peninsula and Iran, is a shallow sea located in the subtropical region. The high salinity (exceeding 39 practical salinity units) and extreme temperatures (35–39°C in summer and 9–11°C in winter) (Riegl and Purkis, 2012) make marine fauna in the Persian Gulf unique and therefore, the region has become the focus of intensive research (Vaughan and Burt, 2016). There are about 55 sponge genera recorded in the Persian Gulf and Actinobacteria have been reported as one of the most abundant bacterial phyla inhabiting sponges of the region (Najafi et al., 2018).

In this work, we report the isolation of Actinobacteria associated with marine sponges collected from two locations from the Persian Gulf, and assessed their potential as producers of antimicrobial agents against a panel of gram-positive and -negative bacteria. The isolates were screened by genotyping to find rare Actinobacteria with the potential to produce novel aromatic polyketides. Further studies with *Streptomonospora* sp. PA3 led to the identification of seven biosynthetic gene clusters from genome sequencing data and isolation of a new bioactive compound denoted persiamycin A. To the best of our knowledge, persiamycin A is only the third secondary metabolite (Metelev et al., 2015; Lee et al., 2016) and the first polyketide that has been discovered from *Streptomonospora*.

## Materials and Methods

### Isolation and Cultivation of Marine Actinomycetes

Marine sponges were collected from Hengam Island (N 26°36′42.54″, E 55°55′28.26″) and Nayband Gulf (N 27°25′55.06″, E 52°38′41.86″) at a depth of 15–20 m, in September 2016, September and October 2017. Sponge samples were washed three times for 5 min at 150 rpm agitation in sterile seawater to remove loosely attached cells and sediments. The washed sponge material was then cut into 1 cm^3^ cubes and homogenized in 10 ml of sterile seawater for 10–15 seconds and then subjected to serial dilutions (10^–1^ to 10^–7^) after which 100 μl was transferred on to agar plates comprising M1 (Mincer et al., 2002), SCA (Starch casein agar) and NA (Nutrient agar) prepared in 1 liter of artificial sea water (26.52 g NaCl, 5.228 g MgCl_2_.6H_2_O, 3.305 g MgSO_4_, 1.141 g CaCl_2_, 0.725 g KCl, 0.202 g NaHCO_3_, 0.083 g NaBr) (Sun et al., 2015). All media were supplemented with nystatin (25 μg/ml) and nalidixic acid (25 μg/ml) to facilitate the isolation of slow-growing Actinobacteria. Nystatin inhibits fungal growth, while nalidixic acid inhibits many fast-growing gram-negative bacteria. The resulting agar plate was incubated at 30°C for 30–60 days. Pure strains of individual colonies were obtained by standard microbiological techniques, and were grown to dense colonies on single agar plates.

For antibacterial assays, a seed culture was prepared from the 45 actinomycetes isolates by inoculating a 250 ml flask containing M1 medium with a single colony of each strain followed by shaking at 200 rpm for 7–10 days at 30°C. The culture filtrates were extracted using ethyl acetate:methanol 1:1 (v/v). The mixture was shaken vigorously for 1 h and the combined organic phases concentrated *in vacuo*. The residual crude extract obtained was dissolved in 2 ml of methanol and preserved at 4°C.

### General DNA Techniques and Microbial Taxonomy

Genomic DNA (gDNA) extraction was performed using the Cinnapure DNA Kit for isolation of DNA from gram-positive bacteria, according to the manufacturer’s protocol. The 16S rRNA was amplified from gDNA by PCR using primers F27 (5′-AGAGTTTGATCCTGGCTCAG-3′) and R1492 (5′-GGTTACCTTGTTACGACTT-3′). The 50 μl PCR mixture contained 20–40 ng gDNA, 20 pmol of each primer, 2.5 U Taq DNA polymerase and 100 μmol of each dNTP and expected PCR product was 1.5 kb (Monciardini, 2002). PCR amplification of conserved minimal polyketide synthase II (PKSII) gene regions was performed using gDNA and a pair of degenerate primers (5′-TSG CST GCT TGG AYG CSA TC-3′) (sense primer) and (5′-TGG AANCCG CCG AAB CCG CT-3′) to amplify a product with the size of 613 bp (Metsä-Ketelä et al., 2002). PCR products were analyzed by electrophoresis on a 1% agarose gel stained with SybrSafe and compared to a 1 kb Plus Ladder (Invitrogen). Purified PCR products were sequenced directly using the same primers that were used for amplification.

For isolation of chromosomal DNA, *Streptomonospora* sp. PA3 was cultured in 30 ml of M1 medium with 0.5% glycine at 30°C for 2 days shaking at 300 rpm. The cells were pelleted and frozen at −20°C. Genomic DNA was extracted using a previously developed protocol (Nikodinovic et al., 2003) with slight modifications. Quality control and the PCR-free shotgun library (Illumina) were prepared at the Finnish Functional Genomics Center (Turku, Finland). A single lane of an Illumina MiSeq v3 sequencer was used to produce 2 × 300 bp reads.

### Antibacterial Assays

Cultures of *Bacillus cereus* (PTCC1816), *Escherichia coli* (PTCC1397), *Klebsiella pneumoniae* (PTCC1290), *Proteus vulgaris* (PTCC1279), *Pseudomonas aeruginosa* (PTCC1310), *Salmonella enterica* (PTCC1787) and *Staphylococcus aureus* (PTCC1337) were grown in nutrient broth (Bacto) and incubated overnight at 37°C. An aliquot (100 μl) of each broth was spread over the surface of a sterile nutrient agar plate. Crude extract from Actinobacteria cultures was added to a sterile paper disc. The plates were then incubated overnight at 37°C, after which the zones of inhibition were recorded. Nalidixic acid (30 μg) and penicillin (20 μg) were used as positive controls against bacteria, while methanol was used as a negative control.

An agar-plate diffusion assay was used to determine the antibacterial activity of purified compound **2**, where 60 μg was applied to a filter disk (6 mm diameter). The test plates were incubated for 24 h at a temperature that permitted optimal growth of the test organisms. The inhibitory activity of **2** on the growth of human tumor cell line from breast carcinoma (MDA-MB-231) was tested by MTT (3-(4,5-dimethylthiazol-2yl)-2,5-diphenyltetrazolium bromide) (Sigma-Aldrich, St. Louis, MO, United States) cell viability assay. MDA-MB-231 cell line cultured in Dulbecco’s Modified Eagle Medium (DMEM) was supplemented with 10% FBS. Cells were grown in triplicates in 12-well microtiter plates at the density of 250 × 10^3^ in DMEM medium containing 10% FBS in a humidified atmosphere of 5.0% CO_2_ in air. Cells (250 × 10^3^ cells/well) were seeded in triplicates in 12-well plates and were cultured overnight. Compound **2** (1, 4, 8, 16, 32, 64, 128, 250, and 500 μg/ml) was added to the cells after incubation for 24 h. Methanol at a final concentration of 0.5% was used as a negative control. After 24-h incubation, MTT analysis of the plates was performed as described earlier (Fotakis and Timbrell, 2006) and IC_50_ was reported as the concentration of extract required for 50% inhibition compared with control cells.

### Computational Analysis

To construct phylogenetic trees, the 16S rRNA and ketosynthase gene sequences were compared to existing sequences in the NCBI database using the BLASTn and BLASTp search programs, respectively. Multiple sequence alignment was performed using ClustalW integrated within the MEGA6.06 software package (Tamura et al., 2013). The gene sequences that encode the ketosynthase α (KSα) subunits involved in polyketide biosynthesis were retrieved from the MIBIG repository (Medema et al., 2015). The neighbor-joining method was employed to construct phylogenetic trees using the default parameters of MEGA 6.06. The reliability of the phylogenetic tree was tested by bootstrap analysis using 1,000 replicates.

For *de novo* assembly of *Streptomonospora* sp. PA3 genome, the quality of the reads were manually checked before and after error correction using FASTQC (v0.11.2) (Andrews, 2010). The reads were assembled using A5-miseq (v20150522) (Coil et al., 2015), contiguated with ABACAS (v1.3.1) (Assefa et al., 2009) using *Streptomyces albus* NK660 (CP007574.1) as the reference, and the gaps were filled using IMAGE (v2.4.1) (Tsai et al., 2010). The final assembly was annotated using RAST (Brettin et al., 2015) and evaluated for completeness using BUSCO (v1.22) (Simão et al., 2015). All programs were used with the default parameters and run on the CSC – IT Center for Science’s Taito super-cluster (Espoo, Finland). The program antiSMASH (Blin et al., 2019) was used for analysis of secondary metabolite pathways and the persiamycin gene cluster was deposited to the MiBiG (Medema et al., 2015) database. For analysis of antimicrobial activities, a heatmap along with a dendrogram was generated using “R” statistical software, with command “pheatmap”. Gene annotations were collected using the SEED viewer of the RAST annotation server (Brettin et al., 2015) using the subcategories cold shock, heat shock and osmotic stress for *Streptomonospora* sp. PA3 and *Streptomyces coelicolor* A3(2), the model *Streptomyces* species (ASM20383v1).

### Production and Purification of Metabolites From Streptomonospora PA3 Strain

For small-scale fermentation, 250-ml flasks containing 100 ml M1 media with artificial sea water were inoculated with *Streptomonospora* sp. PA3. All flasks were cultured on a rotary shaker at 200 rpm and 30°C for 7 days. For large-scale fermentation, pre-cultures (0.1% v/v) were inoculated in a bioreactor (Bioengineering, Type NLF 22, 30 l) containing 20 l M1 medium with artificial sea water at 100 rpm agitation and constant air supply (0.5 bar). After 4 days of cultivation the absorbent (LXA1180, Sunresin, 20 g/l) was added to the bioreactor. After 3 days of additional fermentation, the absorbent was collected, washed with water and finally the compounds bound to the resin were extracted with methanol. Next a liquid-liquid extraction was carried out (methanol:chloroform:Trizmabase (unbuffered) 62.5 mM; 11:82:7). The aqueous phase containing target compounds was washed with chloroform three times. The aqueous phase was concentrated and subsequently subjected to size exclusion chromatography (Sephadex LH-20, GE healthcare) in methanol. Fractions containing a brown and a red compound were concentrated and subjected to preparative HPLC, LC-20 AP with a diode array detector SPD-M20A (Shimadzu) with a reverse-phase column (Phenomenex, Kinetex 5 μm, C18 250 × 10 mm). A mobile phase gradient from 10% acetonitrile containing 18 mM ammonium acetate (pH 3.6) to 100% acetonitrile was used.

### Analysis of Compounds

Analytical HPLC analysis were carried out with a SCL-10Avp HPLC with an SPD-M10Avp diode array detector (Shimadzu) using a reversed-phase column (Phenomenex, Kinetex, 2.6 μm, 4.6 × 100 mm). MS analysis were carried out with either a low resolution MS with a HPLC system (Agilent 1260 Infinity 6120 Quadropole LC/MS) with similar conditions and column as the analytical HPLC or a MicrOTOF-Q high resolution MS with direct injection (Bruker Daltonics).

For NMR analysis, the purified compounds were dried and dissolved in DMSO*-d6* or MeOD (Eurisotop) (**1**) or a mixture of DMSO*-d6* and CDCl_3_ (**2**). The NMR spectra were recorded with a 500 MHz Bruker AVANCE-III NMR-system equipped with a liquid nitrogen cooled Prodigy BBO (CryoProbe) or a 600 MHz Bruker AVANCE-III NMR-system equipped with a liquid nitrogen cooled Prodigy TCI (inverted CryoProbe) at 298 K. The signals were internally referenced to the solvent signals or tetramethylsilane (TMS). The experiments included 1D spectral analysis (^1^H,^13^C) and 2D measurements (COSY, HMBC, and HSQCDE). Topspin (Bruker Biospin) was used for spectral analysis.

### Accession Numbers

The 16S rRNA gene sequences have been deposited in NCBI database with accession numbers MK396795-MK396834 and MK396836-MK396839 and PKSII gene sequence accession numbers MK522047-MK522048, MK5220501, MK522053-MK522061, and MK522063- MK522070. This Whole Genome Shotgun project has been deposited at DDBJ/ENA/GenBank under the accession VTZW00000000. The version described in this paper is version VTZW01000000.

## Results

### Isolation of Actinobacteria

**FIGURE 1:**
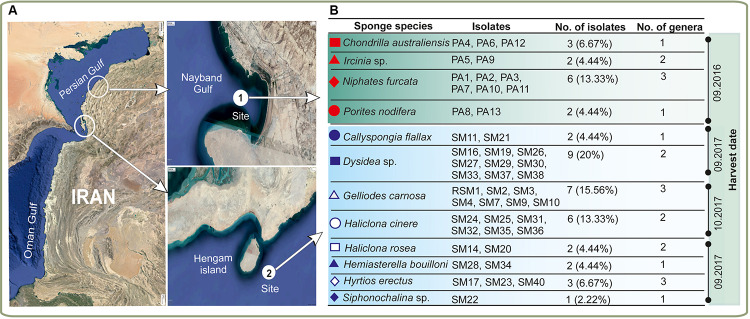
Isolation of sponge-associated actinomycetes. **(A)** Geographical locations of the sampling sites with **(B)** representation of the diversity of the sponge material and actinomycete isolates utilized in this study. The different sponge species are indicated with red and blue symbols. Harvesting of the samples was done in three different times.

Twelve marine sponges were collected from Hengam Island and Nayband Gulf (Figure 1A). Preliminary taxonomic classification based on morphology suggested that all sponges belonged to different species (Figure 1B). In total, 45 unique marine actinobacterial isolates were obtained (Figure 1B) by cultivating homogenized sponge material on three different solid media for 30–60 days. The bacterial strains were cataloged based on colony morphology and formation of various pigments (e.g., brownish white, orange, blackish, white) in aerial hyphae and substrate mycelium. The number of isolates ranged between one and nine bacterial strains per sponge sample, with the maximum number of isolates obtained from *Dysidea* sp.

### Evaluation of Antibacterial Activity

**FIGURE 2:**
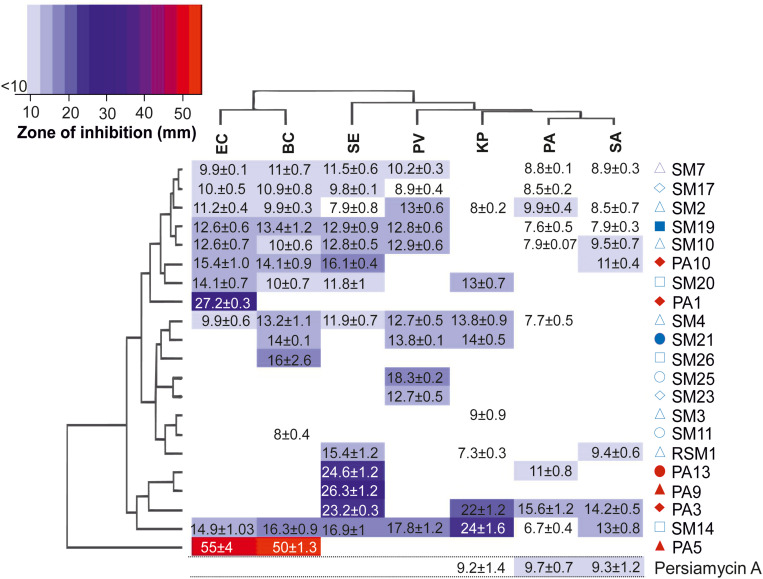
Antibiotic activity of culture extracts. Heatmap diagram illustrating the antimicrobial properties of 22 culture samples probed against seven pathogenic bacterial strains. Colors on the scheme correspond to the diameter of the zone of growth inhibition (mm) in disk diffusion assays. The clustered heat-map reveals two-way display of antibacterial data matrix between the different isolates and the pathogens tested. The hierarchical cluster analyses of the rows and columns determine the relationships of the isolates and the pathogens based on the observed results, respectively. Legend: PV, *P. vulgaris*; BC, *B. cereus*; KP, *K. pneumoniae*; EC, *E. coli*; SE, *S. enterica*; PA, *P. aeruginosa*, and SA, *S. aureus*. The red and blue symbols adjacent to the isolated bacterial strains correspond to sponge host (Figure 1) from which the strain was isolated. Activity of persiamycin A was tested with pure isolated compound.

Extracts from all bacterial isolates were evaluated for their antimicrobial potency against seven bacterial pathogens (*Staphylococcus aureus*, *Bacillus cereus*, *Pseudomonas aeruginosa*, *Proteus vulgaris*, *Salmonella enterica*, *Klebsiella pneumonia*, and *Escherichia coli*). Out of 45 isolates, 22 isolates (50%) showed positive bioactivity, with two isolates (SM2 and SM14) demonstrating significant antibacterial activity against all tested pathogens (Figure 2). In contrast, isolates SM25 and PA9 were highly specific with considerable growth inhibitory activity only against *P. vulgaris* (18.3 mm) and *S. enterica* (26.3 mm), respectively. Isolate PA5 exhibited maximum antibacterial potential against *B. cereus* (55 mm) and *E. coli* (50 mm). Isolate PA3 displayed the highest growth inhibition against *S. aureus* (14.2 mm) and *P. aeruginosa* (15.6 mm).

### Identification of Bacterial Strains by Amplification of 16S rRNA Genes and Phylogenetic Analysis

**FIGURE 3:**
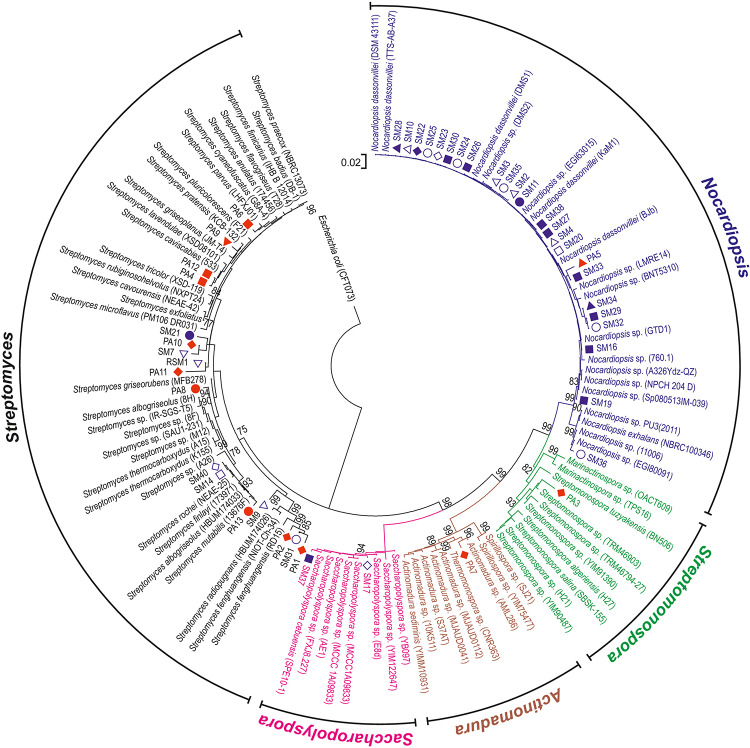
Phylogenetic analysis of actinomycete isolates. The phylogenetic tree was constructed from 16S rRNA gene sequences from sponge isolates together with type strain sequences obtained from sequence databases. The tree was rooted using the sequence from *E. coli* as an outgroup. Bootstrap values greater than 75% are shown at the nodes and are based on 1000 replicates. Scale bar indicates 0.02 substitutions per nucleotide. The red and blue symbols adjacent to the analyzed bacterial strains correspond to sponge host (Figure 1).

All actinobacterial isolates were identified based on 16S rRNA gene sequences, which revealed that the 45 strains exhibited a high level of sequence similarity (96–100%) with existing strains in databases (Figure 3). The strains were divided into five families and six genera. Most of the isolates were grouped into *Nocardiopsis* (53%) and *Streptomyces* (38%), whereas the minority belonged to rare actinomycetes. These included genera from *Pseudonocardia* family such as *Saccharopolyspora* (2%) and *Amycolatopsis* (2%), *Streptomonospora* (2%) and *Actinomadura* (2%). The phylogenetic analysis demonstrated that most isolates from the genus *Nocardiopsis* formed a major clade, along with most closely related type strains recovered from databases. Similarly, *Streptomonospora* and *Actinomadura* isolates were closely related to their type strains under bootstrap values of 96 and 62%, respectively. The genus *Streptomyces* formed three closely related clades under bootstrap support values of 99, 97, and 70%.

### Probing the Genomic Potential of Actinomycete Isolates for Production of Aromatic Polyketides

**FIGURE 4:**
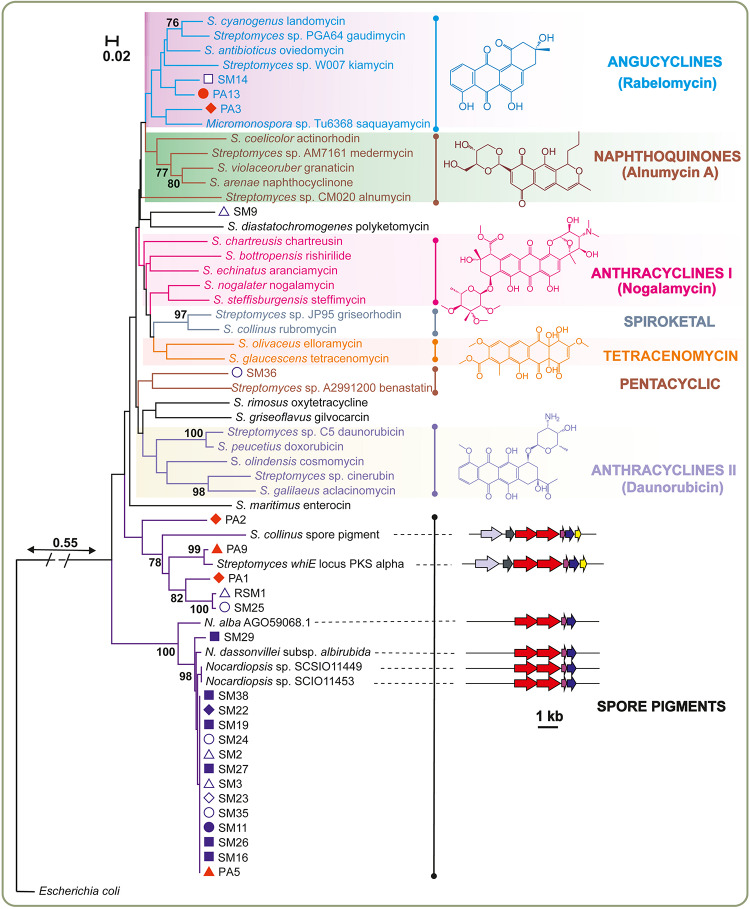
Genotyping the potential of sponge-associated Actinobacteria for production of aromatic polyketides. The neighbor-joining tree was constructed using aligned KSα domain amino acid sequences. Next to the KSα gene name, the identified or predicted compounds of the gene cluster are indicated. Bootstrap values calculated from 1000 replicates are shown at the respective nodes when the calculated values were 75% or greater. The scale bar represents 0.02 substitutions per amino acid position. The red and blue symbols adjacent to the analyzed bacterial strains correspond to sponge host (Figure 1). The boundary between spore pigment and antibiotic biosynthesis sequences was assessed based on additional analysis of gene cluster contents. Verified spore pigment gene clusters from *Streptomyces* displayed similarity and synteny to unknown biosynthetic gene clusters from *Nocardiopsis* type strains with conservation of ketosynthases (red), acyl carrier proteins (pink) and cyclases (blue).

The marine *Streptomyces* species and rare actinomycete isolates were probed for the presence of ketosynthase α (KSα) genes, which together with the ketosynthase β subunit are essential genes in the biosynthesis of aromatic polyketides (Metsä-Ketelä et al., 2002). KSα genes could be amplified and sequenced from 23 isolates with degenerate PCR primers. A phylogenetic tree (Figure 4) was constructed from these sequences together with experimentally characterized sequences retrieved from the MIBiG database (Medema et al., 2015). The results demonstrated that KSα genes involved in antibiotic biosynthesis were scarce, since the majority of sequences clustered with sequences responsible for formation of biosynthetically related spore pigments. Four sequences were grouped with the *whiE* ORF III from *S. coelicolor*, which has been confirmed to be involved in spore pigment formation (Shen et al., 1999). Fourteen sequences mainly from *Nocardiopsis* formed a highly conserved clade between *Streptomyces* spore pigments and genes involved in antibiotic biosynthesis. However, since spore pigment formation has not been studied in *Nocardiopsis*, we extended our sequence analysis to encompass the surrounding genomic *loci* in the reference strains. Bioinformatic analysis of the putative spore pigment gene clusters from *Nocardiopsis* and *Streptomyces* by MultiGeneBlast (Medema et al., 2013) revealed a set of four genes (minimal PKS and a cyclase) in *Nocardiopsis* that also reside in the *whiE* locus from *S. coelicolor* (Figure 4). The data suggests that these highly conserved sequences from marine *Nocardiopsis* may be involved in spore pigment formation. The phylogenetic analysis revealed only five sequences, which could be classified unambiguously as ketosynthases involved in antibiotic biosynthesis. Three of these originated from *Streptomyces* sp. (SM9, SM14 and PA13) and two from rare Actinobacteria (*Streptomonospora* sp. PA3 and *Nocardiopsis* sp. SM36) (Figure 4).

### Discovery of Persiamycin A From *Streptomonospora* sp. PA3

The isolate *Streptomonospora* sp. PA3 was selected for further studies based on the following criteria: (i) the bioactivity of the crude extract against *S. aureus* and *P. aeruginosa* differed from the other strains (Figure 2), (ii) the strain belonged to rare actinomycetes genera (Figure 3) underexplored for their potential for production of natural products and (iii) a KSα gene predicted to be involved in antibiotic biosynthesis was detected from the genome (Figure 4).

**FIGURE 5:**
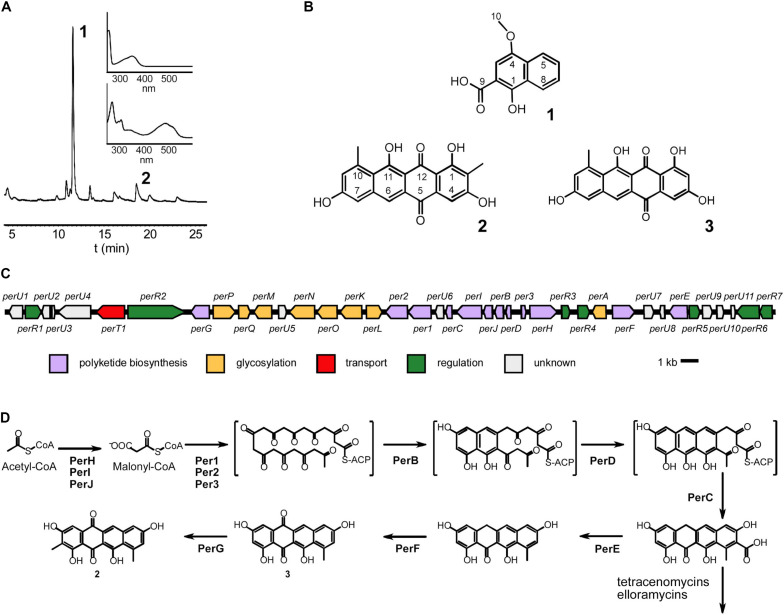
Characterization of *Streptomonospora* sp. PA3 metabolites and biosynthesis of persiamycin A. **(A)** HPLC trace of a methanol extract shown at 256 nm and the UV/VIS spectra of the purified compounds 1-hydroxy-4-methoxy-2-naphthoic acid **(1)** and persiamycin A **(2)**. **(B)** Chemical structures of **(1)**, **(2)** and the related compound tetracenomycin D **(3)**. **(C)** Graphical representation of the persiamycin gene cluster and **(D)** model for the biosynthesis of **2**. The biosynthetic pathways of persiamycins and tetracenomycin/elloramycins appear to have diverged after fourth ring cyclization.

The strain was grown in large scale in a bioreactor and two main metabolites (Figures 5A,B) were purified using several chromatographic techniques. The first beige compound (**1**) was identified by NMR (Figure 5B, Supplementary Material) and MS, (ESI) *m/z*: [M-H]^–^ calc. for C_12_H_9_O_4_^–^ 217.1; found 217.1) as 1-hydroxy-4-methoxy-2-naphthoic acid. The second red compound (**2**) was analyzed by NMR (Figure 5B, Supplementary Material). The proton spectrum shows four broad signals in the aromatic region (δ 6.98–8.08), two methyl groups and three partially exchanged hydroxyl groups, one hydroxyl group is completely exchanged. The HSQCDE analysis shows that one of the methyl groups has unusually upfield carbon signal (δ 9.2) that indicates that the methyl group could be surrounded by hydroxyl groups. This finding was supported by HMBC analysis, as the methyl hydrogens (δ 2.27) have HMBC correlation to phenolic carbons (δ 158.0, 161.8). The second methyl group has a more canonical shift (δ 24.8). The positioning of the methyl group is supported by HMBC signals to C10a and from C9 to the methyl group. Furthermore, the position of the quinone is supported by HMBC signals from both sides of the entity. The HR-MS result [(ESI) *m/z*: [M-H]^–^: found 349.0706; calc. for C_20_H_13_O_6_^–^ 349.0718] supports the NMR structural elucidation, which indicates that compound **2** is a new aromatic polyketide that was denoted as persiamycin A. The antimicrobial activity of **2** was determined by agar plate diffusion assays, which demonstrated that the compound exhibited a moderate growth inhibition of gram-positive bacterium *S. aureus* and gram-negative bacteria *K. pneumoniae and P. aeruginosa* (Figure 2). In order to test cytotoxicity and the therapeutic potential of **2**, the antiproliferative potency was assayed against breast carcinoma MDA-MB-231 tumor cell lines, which showed weak growth inhibitory activity with an IC_50_ value of 250 μg/ml.

### Genome Sequencing of *Streptomonospora* sp. PA3

Next we acquired the draft genome sequence of *Streptomonospora* sp. PA3 by Illumina MiSeq, which was assembled *de novo* into 24 scaffolds with an N50 of 575,293 bp. The final genome assembly was 5.7 Mbp with a GC content of 72.9% and a median coverage of 292×. BUSCO analysis searched for 40 single-copy orthologs and found 40 (100%) were complete, one was found multiple times throughout the assembly and none were identified as fragmented. Sequence analysis with antiSMASH (Blin et al., 2019) revealed that the genome contained only seven gene clusters potentially involved in secondary metabolism. Only one aromatic polyketide gene cluster was observed (Figure 5C), which harbored 12 and 8 genes putatively involved in polyketide assembly and glycosylation (Table 1), respectively. This included *perHIJ* for formation of malonyl-CoA by an acetyl-CoA carboxylase complex and the minimal polyketide synthase *per123* responsible for synthesis of the polyketide backbone (Figure 5D). In addition, a dedicated set of aromatases/cyclases *perABCD*, resembling those found on the tetracenomycin biosynthetic pathways such as TcmI (Thompson et al., 2004), TcmJ (McDaniel et al., 1995) and TcmN (Ames et al., 2008) were discovered (Figure 5D). Three genes *perEFG* were identified for post-PKS tailoring steps, which could finalize the formation of the chromophore of **2** (Figure 5D). Seven genes involved in regulation of the pathways could be observed and a gene homologous to major facilitator superfamily transporters. The remaining six other gene clusters could be classified to belong to families of terpene (two), lantipeptide (one), modular type I polyketide (one), non-ribosomal peptide synthetase (one) and hybrid polyketide-peptide metabolites, but no metabolites associated with these pathways could be observed from culture extracts.

**TABLE 1:** Deduced functions of the gene products residing in the persiamycin cluster.

**Gene product**	**RAST annotation**	**Size (AA)**	**Most similar ORF [species] (identity %)**	**Accession no.**
PerU1	3-oxoacyl-[acyl-carrier protein] reductase	255	MULTISPECIES: SDR family oxidoreductase [*Streptomyces*](90)	WP_043485899
PerR1	LysR-type transcriptional regulator	297	LysR family transcriptional regulator [*Nocardiopsis* sp. JB363] (84)	WP_087099906.1
PerU2	hypothetical protein	149	hypothetical protein [*Nocardiopsis sinuspersici*] (77)	WP_077691354.1
PerU3	hypothetical protein	62	2-isopropylmalate synthase [*Clostridium asparagiforme*] (39)	WP_040410861.1
PerU4	glucoamylase	608	glycoside hydrolase family 15 protein [*Amycolatopsis taiwanensis*] (80)	WP_027940885.1
PerT1	major facilitator superfamily MFS_1	526	MFS transporter [*Nocardiopsis halophila*] (69)	WP_051063540.1
PerR2	signal transduction response regulator	1074	AAA family ATPase [*Nocardiopsis halophila*] (58)	WP_017537044.1
PerG	O-methyltransferase	342	class I SAM-dependent methyltransferase [*Nonomuraea* sp. KC333] (71)	WP_111179428.1
PerP	NDP-hexose 3-C-methyltransferase TylCIII	412	class I SAM-dependent methyltransferase [*Nonomuraea* sp. KC333] (76)	WP_111179448.1
PerQ	dTDP-4-dehydrorhamnose 3,5-epimerase	217	dTDP-4-keto-6-deoxy-D-glucose epimerase [*Nonomuraea* sp. KC333] (72)	WP_111179449.1
PerM	dTDP-glucose 4,6-dehydratase	327	dTDP-glucose 4,6-dehydratase [*Nonomuraea* sp. KC333] (74)	WP_111179429.1
PerU5	hypothetical protein	147	hypothetical protein [*Nonomuraea* sp. KC333] (60)	WP_111179451.1
PerN	hypothetical protein	467	NDP-hexose 2,3-dehydratase [*Nonomuraea* sp. KC333] (67)	WP_111179427.1
PerO	aminotransferase	373	DegT/DnrJ/EryC1/StrS family aminotransferase [*Nonomuraea* sp. KC333] (78)	WP_111179426.1
PerK	glycosyltransferase	404	DUF1205 domain-containing protein [*Nonomuraea* sp. KC333] (69)	WP_111179425.1
PerL	glucose-1-phosphate thymidynyltransferase	300	glucose-1-phosphate thymidylyltransferase [*Nonomuraea* sp. KC333] (80)	WP_111179424.1
Per2	polyketide chain length factor WhiE-CLF paralog	408	ketosynthase chain-length factor [*Nonomuraea* sp. KC333] (74)	WP_111179437.1
Per1	polyketide beta-ketoacyl synthase WhiE-KS paralog	425	beta-ketoacyl-[acyl-carrier-protein] synthase family protein [*Nonomuraea* sp. KC333] (82)	WP_111179436.1
PerU6	hypothetical protein	151	DUF1772 domain-containing protein [*Nonomuraea* sp. KC333] (66)	WP_111179435.1
PerC	polyketide cyclase WhiE VII	107	TcmI family type II polyketide cyclase [*Nonomuraea* sp. KC333] (75)	WP_111179434.1
PerI	biotin carboxylase	465	acetyl-CoA carboxylase biotin carboxylase subunit [*Catenulispora acidiphila*] (64)	WP_012785313.1
PerJ	biotin carboxyl carrier protein of acetyl-CoA carboxylase	164	acetyl-CoA carboxylase biotin carboxyl carrier protein [*Streptomyces thermoautotrophicus*] (49)	WP_066890277.1
PerB	aromatase WhiE VI	162	polyketide cyclase [*Nonomuraea* sp. KC333] (72)	WP_111179433.1
PerD	polyketide cyclase WhiE II	142	cupin domain-containing protein [*Nonomuraea* sp. KC333] (70)	WP_111179432.1
Per3	acyl carrier protein	85	acyl carrier protein [*Nonomuraea* sp. KC333] (67)	WP_111179418.1
PerH	acetyl-CoA carboxyl transferase	507	acetyl-CoA carboxylase carboxyl transferase subunit alpha [*Nonomuraea* sp. KC333] (72)	WP_111179419.1
PerR3	transcriptional regulator MarR	154	DNA-binding transcriptional regulator, MarR family [*Thermostaphylospora chromogena*] (72)	SDQ92350.1
PerR4	regulatory protein	218	AfsR/SARP family transcriptional regulator [*Nonomuraea* sp. KC333] (68)	WP_111179423.1
PerA	3-oxoacyl-ACP reductase	261	SDR family oxidoreductase [*Nonomuraea* sp. KC333] (78)	WP_111179422.1
PerF	FAD binding protein	415	NAD(P)/FAD-dependent oxidoreductase [*Nonomuraea* sp. KC333] (75)	WP_111179417.1
PerU7	oxidoreductase	180	NAD-dependent epimerase/dehydratase family protein [*Nonomuraea* sp. KC333] (71)	WP_111179431.1
PerU8	hypothetical protein	93	antitoxin [*Nocardiopsis baichengensis*] (68)	WP_017558586.1
PerE	carboxylesterase NP	339	alpha/beta hydrolase [*Tsukamurella* sp. 1534] (76)	WP_081586088.1
PerR5	transcriptional regulator TetR-family	199	TetR/AcrR family transcriptional regulator [*Brevibacterium aurantiacum*] (63)	WP_009884360.1
PerU9	bifunctional deaminase-reductase domain protein	184	deaminase [*Streptomyces* sp. KM273126] (83)	WP_131570927.1
PerU10	hypothetical protein	132	hypothetical protein [*Streptomonospora alba*] (84)	WP_040271289.1
PerU11	pterin-4-alpha-carbinolamine dehydratase	75	4a-hydroxytetrahydrobiopterin dehydratase [*Haloactinopolyspora alba*](80)	WP_106538811.1
PerR6	putative two-component system sensor kinase	413	HAMP domain-containing protein [*Streptomonospora alba*] (93)	WP_040274336.1
PerR7	putative two-component system response regulator	229	response regulator transcription factor [*Streptomonospora alba*] (96)	WP_040274337.1

*Streptomonospora* sp. PA3 was isolated from a unique environment that includes wide fluctuations in annual temperatures, high temperature maximum and high salinity. We compared the number of genes related to cold shock, heat shock and osmotic stress to the model *Streptomyces coelicolor* A3(2) from New Brunswick, NJ, United States (Kutzner and Waksman, 1959). We found the number of genes involved in cold and heat shock to be comparable between the two strains (Table 2), relative to their genome sizes of 5.7 and 8.7 Mbp, respectively. However, *Streptomonospora* sp. PA3 has 25 genes related to osmotic stress response in comparison to only 17 genes in *Streptomyces coelicolor* A3(2) (Table 2), which reflects the environment of each isolate with high marine or low terrestrial salinity, respectively.

**TABLE 2:** Environmental stress-related genes in *Steptomonospora* sp. PA3 vs S*treptomyces coelicolor* A3(2).

**Feature in subsystems**	**Role**	**PA3 copy no.**	**A3(2) copy no.**
Cold shock	Cold shock protein CspA	3	2
	Cold shock protein CspC	1	1
	Cold shock protein CspD	0	1
	Cold shock protein CspG	0	1
Heat shock	Radical SAM family enzyme in heat shock gene cluster	1	1
	Heat-inducible transcription repressor HrcA	1	1
	Transcriptional repressor of DnaK operon HspR	2	3
	Chaperone protein DnaK	1	1
	Chaperone protein DnaJ	2	2
	Ribonuclease PH	1	1
	tmRNA-binding protein SmpB	1	1
	Heat shock protein GrpE	1	1
	Translation elongation factor LepA	1	1
	Ribosome-associated heat shock protein	1	1
	Nucleoside 5-triphosphatase RdgB	1	1
	rRNA small subunit methyltransferase E	1	1
	rRNA small subunit methyltransferase I	1	1
Osmotic stress	Glycerol uptake facilitator protein	1	2
	Diaminobutyrate-pyruvate aminotransferase	1	1
	Ectoine hydroxylase	1	1
	L-ectoine synthase	1	1
	L-2,4-diaminobutyric acid acetyltransferase	1	1
	Sarcosine *N*-methyltransferase	1	0
	Glycine *N*-methyltransferase	1	0
	Dimethylglycine N-methyltransferase	1	0
	L-proline glycine betaine ABC transport system permease protein ProV	3	3
	Glycine betaine ABC transport system, permease/glycine betaine-binding protein OpuABC	1	1
	Glycine betaine ABC transport system permease protein	1	1
	Glycine betaine transport system OpuAA	0	1
	Glycine betaine transport system OpuAB	0	1
	L-proline glycine betaine binding ABC transporter protein ProX	2	1
	L-proline glycine betaine ABC transport system permease protein ProW	3	0
	Betaine aldehyde dehydrogenase	1	1
	Choline-sulfatase	1	0
	Choline dehydrogenase	2	1
	High-affinity choline uptake protein BetT	4	1

## Discussion

Among all the bacteria-sponge associations, Actinobacteria have been shown to comprise a large section of the microbial community in the marine symbiota (Abdelmohsen et al., 2014). Studies on the Actinobacteria-associated marine sponges have identified at least 28 recognized genera of actinomycetes (Taylor et al., 2007), with the dominant species belonging to the genera *Streptomyces* and *Mycobacterium* (Seipke et al., 2012). Another study reported that *Streptomyces* sp. and *Micrococcus* sp. were the most common actinomycete genera from Mauritius sponges (Beepat et al., 2004). Interestingly, out of the 45 strains isolated from marine sponges in the current study, 24 (53%) isolates belonged to *Nocardia* and 17 (38%) to *Streptomyces*.

In total, species from four rare genera (*Actinomadura*, *Amycolatopsis*, *Saccharopolyspora*, and *Streptomonospora*) were isolated from the Persian Gulf marine sponges for the first time. *Streptomonospora* are a group of extreme halophilic filamentous actinomycetes of the family *Nocardiopsaceae*, which form a distinct branch in the 16S rRNA phylogenetic tree closest to the genera *Nocardiopsis* and *Thermobifida* (Cai et al., 2008). *Streptomonospora* have previously been detected in hypersaline soil (Cai et al., 2008) and from a salt lake (Cai et al., 2009). The isolation of *Streptomonospora* sp. PA3 here and previous detection of few *Streptomonospora* in marine sediments and sponges (Zhang et al., 2013; Sun et al., 2015) indicates that this genus also habitats the marine environment. Actinobacterial genera such as *Actinomadura*, *Micromonospora*, and *Streptosporangium* are thermotolerants which survive at 50°C (Kurapova et al., 2012), which account for the isolation of *Actinomadura* sp. PA7 from the subtropical sampling site. *Nocardia*, *Nocardiopsis*, and *Saccharopolyspora* have been identified from land around the Sambhar Salt Lake, Arabian Sea and from a saltern beside the Bay of Bengal (Mohammadipanah and Wink, 2016), hypersalinic locations geographically relevant to the isolation site of *Saccharopolyspora* sp. SM17 and SM37 presented here. Sequencing of the genome of *Streptomonaspora* sp. PA3 provided insight into the survival of the species under high osmotic stress (Table 2). Comparative analysis against the genome of the terrestrial model Actinobacteria, *S. coelicolor* A3(2), revealed that while gene products for the utilization of the osmolyte L-ectoine (da Costa et al., 1998; Czech et al., 2018) were similar, *Streptomonospora* sp. PA3 appears to have an increased capacity for the uptake and synthesis of betaine-type (da Costa et al., 1998; Zou et al., 2016) osmoregulatory compounds. In particular, four gene products for the uptake of choline were detected, which could feasibly be converted to trimethylglycine by choline dehydrogenase and betaine aldehyde dehydrogenase (Table 2). Interestingly, *Streptomonospora* sp. PA3 may possibly encode a second pathway, which is not present in *S. coelicolor* A3(2), to produce betaine through sequential methylation of glycine by glycine *N*-methyltransferase and dimethylglycine *N*-methyltransferase (Table 2).

The characterizations of rare microbial genera and new species have been proposed to be necessary to meet the demands for the continuing search for novel biologically active compounds (Metelev et al., 2015). Studies have shown that sponge-associated actinomycetes might represent an unexplored resource for pharmaceutical drug discovery (Montalvo et al., 2005). Here we focused on aromatic polyketide natural products, since to date these have been isolated from a rather narrow range of species. In particular, the majority of these so-called type II polyketides (e.g., tetracycline, doxorubicin, actinorhodin and rabelomycin) (Hertweck et al., 2007) have been obtained from terrestrial *Streptomyces* species, which appear to contain on average 0–2 gene clusters responsible for production of these metabolites (Metsä-Ketelä et al., 2002). This is in stark contrast to type I polyketides (e.g., erythromycin) and non-ribosomal peptides (e.g., vancomycin and penicillin), which can be widely found from diverse microbial phyla (e.g., Actinobacteria, Cyanobacteria and Firmicutes) (Siezen and Khayatt, 2008; Mahajan and Balachandran, 2012). Our genotyping of sponge-associated Actinobacteria supported the previous assessment, since only one gene responsible for the synthesis of aromatic polyketide antibiotics could be found from a rare actinomycete, *Streptomonospora* sp. PA3, out of the 45 isolates analyzed here (Figure 4). The key source of antibiotic KSα gene sequences was marine *Streptomyces* sp., where antibiotic biosynthesis genes could be detected only in approximately one fourth of the isolates (4 out of 17). This represents a significant decrease in comparison to genotyping of terrestrial *Streptomyces*, in which KSα genes were found in nearly two thirds (54 out of 87 isolates) (Metsä-Ketelä et al., 2002). Three sequences were predicted to encode proteins responsible for the biosynthesis of angucycline metabolites (Figure 4), which belong to a class of aromatic polyketides that have previously been isolated from marine *Streptomyces* species (Qu et al., 2019). Surprisingly, the sequence from *Streptomonospora* sp. PA3 also resided in the angucycline clade instead of grouping together with tetracenomycin-type sequences, which may reflect the divergence of *Streptomonospora* genus from reference *Streptomyces* strains. This implies that the scarcity of sequences from rare Actinobacteria (Figure 4), where the functions of the genes have been experimentally confirmed, needs to be addressed in the future to fully comprehend the evolutionary relationships of aromatic type II polyketides.

The observation of a KSα gene in *Streptomonospora* sp. PA3 was noteworthy, since to the best of our knowledge no polyketides have previously been isolated from this genus. In effect, only two secondary metabolites, streptomonomicin lasso peptides (Metelev et al., 2015) and marinopyrone α-pyrones (Lee et al., 2016), have been characterized from *Streptomonospora*. Moreover, since *Streptomonospora* sp. PA3 represented potentially a new strain and crude extracts from the strain displayed significant antimicrobial activity, the biosynthetic capacity of the strain was studied in more detail. The work confirmed that the red-pigmented compound detected in culture extracts was a new aromatic polyketide persiamycin A (**2**), which has a chemical structure similar to tetracenomycin D (**3**) (Figure 5B) isolated from *S. glaucescens* (Rohr et al., 1988). Compound **2** was found to have moderate antibacterial activity toward *S. aureus*, *K. pneumoniae* and *P. aeruginosa* (Figure 2) and weak potency (IC_50_: 715 μM) against breast carcinoma (MDA-MB-231) cell line. It is noteworthy that the cytotoxicity was approximately 30-fold lower that what has been reported for tetracenomycin D against L1210 leukemia cells (Martin et al., 2002).

The second metabolite identified from cultures of *Streptomonospora* sp. PA3 was identified as 1-hydroxy-4-methoxy-2-naphthoic acid, a compound previously isolated from *Streptosporangium cinnabarinum* ATCC 31213 (Pfefferle et al., 1997). Non-methylated 1,4-dihydroxy-2-naphthoic acids are involved in biosynthetic pathways of menaquinones, which are important constituents of respiratory electron transport chain. Moreover it has been shown that the non-methylated intermediate contributes to the survival of *Listeria monocytogenes* in the absence of menaquinones (Chen et al., 2017).

Genome mining has been widely used in the identification of novel metabolites in terrestrial actinomycetes, in particular in *Streptomyces* (Belknap et al., 2020) and to a lesser extent in marine actinomycetes (Guerrero-Garzón et al., 2020; Lee et al., 2020). Recent examples include the discovery of neaumycin B (Kim et al., 2018) and new tunicamycins (Xu D. et al., 2018) from a marine-derived *Micromonospora* and *Streptomyces* sp. DUT11, respectively. Genome sequencing of *Streptomonospora* sp. PA3 revealed a limited set of seven biosynthetic gene clusters for production of secondary metabolites, which contrasts the 20–40 gene clusters typically associated with terrestrial *Streptomyces* (Baral et al., 2018). One gene cluster harbored several genes related to tetracenomycin biosynthesis and was therefore most likely responsible for formation of **2**. Bioinformatics analysis indicated that the biosynthesis could be initiated through formation and attachment of a decaketide to the acyl-carrier protein Per3 by the ketosynthase α (Per1) and β (Per2) heterodimer (Figure 5D). The cyclases PerB, PerC and PerC that were all related to proteins found on the tetracenomycin (Ames et al., 2008) and elloramycin (Ramos et al., 2008) pathways could shape the cyclization process through a C7–C14 folding pattern. The biosynthesis of **2** appears to diverge from the tetracenomycin and elloramycin pathways during the tailoring steps, which may explain how the novel chemistry has evolved. Most tetracenomycins and all elloramycins have retained the polyketide-derived carboxy functional group at C9 (Rohr et al., 1988; Ramos et al., 2008), but the persiamycin pathway encodes a α/β-hydrolase PerE that could plausibly catalyze decarboxylation. Similarly, quinone formation proceeds via co-factor independent mono-oxygenases (Sciara et al., 2003) in tetracenomycin and elloramycin biosynthesis, whereas the flavoenzyme PerF related to angucycline mono-oxygenases (Koskiniemi et al., 2007) is the most likely candidate for this step in persiamycin biosynthesis. Compound **3** is considered a shunt product from the tetracenomycin pathway (Yue et al., 1986), but likely a true intermediate toward persiamycins (Figure 5). All of these pathways encode methyltransferases, but whereas tetracenomycins and elloramycins are *O*-methylated, the chemically more challenging *C*-methylation could be catalyzed by PerG in the biosynthesis of **2**. The persiamycin cluster also harbors genes for biosynthesis and transfer of an aminosugar, but glycosylated persiamycin metabolites could not be characterized from laboratory cultures.

In conclusion, the results presented here suggest that marine actinomycetes from Persian Gulf sponges are an underexplored source of rare species, which have the potential to provide new biosynthetic pathways and to produce novel bioactive natural products. Even though *Streptomonospora* do not appear to contain as many biosynthetic gene clusters as terrestrial *Streptomyces*, these pathways seem to have evolved and diversified toward a distinct chemical space. Our work also reinforces the efficiency of genotyping as a tool to guide the microbial drug discovery pipeline toward bioactive compounds.

## Data Availability Statement

The datasets generated for this study can be found in the NCBI GenBank with the accession number VTZW00000000.

## Author Contributions

SM and MM-K designed research and wrote the manuscript. SM, VS, BB, KY, and AA performed the experiments. SM, VS, BB, KY, AA, and MM-K analyzed the data.

## Conflict of Interest

The authors declare that the research was conducted in the absence of any commercial or financial relationships that could be construed as a potential conflict of interest.

## References

[B1] Abdelmohsen U. R. Bayer K. Hentschel U. (2014). Diversity, abundance and natural products of marine sponge-associated actinomycetes. *Nat. Prod. Rep.* 31 381–399. 10.1039/c3np70111e 24496105

[B2] Achlatis M. Pernice M. Green K. de Goeij J. M. Guagliardo P. Kilburn M. R. (2019). Single-cell visualization indicates direct role of sponge host in uptake of dissolved organic matter. *Proc. R. Soc. B*. 286:20192153. 10.1098/rspb.2019.2153 31795848 PMC6939258

[B3] Ames B. D. Korman T. P. Zhang W. Smith P. Vu T. Tang Y. (2008). Crystal structure and functional analysis of tetracenomycin ARO/CYC: implications for cyclization specificity of aromatic polyketides. *Proc. Natl. Acad. Sci. U. S. A.* 105 5349–5354. 10.1073/pnas.0709223105 18388203 PMC2291100

[B4] Andrews S. (2010). *FastQC: A Quality Control Tool for High Throughput Sequence Data.* Available online at: https://www.bioinformatics.babraham.ac.uk/projects/fastqc (accessed October 6, 2011).

[B5] Assefa S. Keane T. M. Otto T. D. Newbold C. Berriman M. (2009). ABACAS: algorithm-based automatic contiguation of assembled sequences. *Bioinformatics* 25 1968–1969. 10.1093/bioinformatics/btp347 19497936 PMC2712343

[B6] Baral B. Akhgari A. Metsä-Ketelä M. (2018). Activation of microbial secondary metabolic pathways: avenues and challenges. *Synth. Syst. Biotechnol.* 3 163–178. 10.1016/j.synbio.2018.09.001 30345402 PMC6190515

[B7] Beepat S. S. Appadoo C. Marie D. E. P. Sadally S. B. Paula J. P. M. Sivakumar K. (2004). First records of sponge-associated actinomycetes from two coastal sponges from Mauritius. *West Indian Ocean J. Mar. Sci.* 15 31–38.

[B8] Belknap K. C. Park C. J. Barth B. M. Andam C. P. (2020). Genome mining of biosynthetic and chemotherapeutic gene clusters in *Streptomyces* bacteria. *Sci. Rep.* 10 2003–2011. 10.1038/s41598-020-58904-9 32029878 PMC7005152

[B9] Blin K. Shaw S. Steinke K. Villebro R. Ziemert N. Lee S. Y. (2019). antiSMASH 5.0: updates to the secondary metabolite genome mining pipeline. *Nucleic Acids Res.* 47 W81–W87. 10.1093/nar/gkz310 31032519 PMC6602434

[B10] Brettin T. Davis J. J. Disz T. Edwards R. A. Gerdes S. Olsen G. J. (2015). RASTtk: a modular and extensible implementation of the RAST algorithm for building custom annotation pipelines and annotating batches of genomes. *Sci. Rep.* 5:8365. 10.1038/srep08365 25666585 PMC4322359

[B11] Bull A. T. Stach J. E. M. (2007). Marine actinobacteria: new opportunities for natural product search and discovery. *Trends Microbiol.* 15 491–499. 10.1016/j.tim.2007.10.004 17997312

[B12] Cai M. Tang S. K. Chen Y. G. Li Y. Zhang Y. Q. Li W. J. (2009). *Streptomonospora amylolytica* sp. nov. and Streptomonospora flavalba sp. nov., two novel halophilic actinomycetes isolated from a salt lake. *Int. J. Syst. Evol. Microbiol.* 59 2471–2475. 10.1099/ijs.0.007682-0 19622663

[B13] Cai M. Zhi X. Y. Tang S. K. Zhang Y. Q. Xu L. H. Li W. J. (2008). *Streptomonospora halophila* sp. nov., a halophilic actinomycete isolated from a hypersaline soil. *Int. J. Syst. Evol. Microbiol.* 58 1556–1560. 10.1099/ijs.0.65513-0 18599694

[B14] Carroll A. R. Copp B. R. Davis R. A. Keyzers R. A. Prinsep M. R. (2019). Marine natural products. *Nat. Prod. Rep.* 36 122–173. 10.1039/C8NP00092A 30663727

[B15] Chen G. Y. McDougal C. E. D’Antonio M. A. Portman J. L. Sauer J. D. (2017). A genetic screen reveals that synthesis of 1,4-dihydroxy-2-naphthoate (DHNA), but not full-length menaquinone, is required for Listeria monocytogenes cytosolic survival. *mBio* 8:e00119-17. 10.1128/mBio.00119-17 28325762 PMC5362031

[B16] Coil D. Jospin G. Darling A. E. (2015). A5-miseq: an updated pipeline to assemble microbial genomes from Illumina MiSeq data. *Bioinformatics* 31 587–589. 10.1093/bioinformatics/btu661 25338718

[B17] Czech L. Poehl S. Hub P. Stöveken N. Bremer E. (2018). Tinkering with osmotically controlled transcription allows enhanced production and excretion of ectoine and hydroxyectoine from a microbial cell factory. *Appl. Environ. Microbiol.* 84:e01772-17. 10.1128/AEM.01772-17 29101191 PMC5752866

[B18] da Costa M. S. Santos H. Galinski E. A. (1998). An overview of the role and diversity of compatible solutes in Bacteria and Archaea. *Adv. Biochem. Eng. Biotechnol.* 61 117–153. 10.1007/bfb0102291 9670799

[B19] Fotakis G. Timbrell J. A. (2006). In vitro cytotoxicity assays: comparison of LDH, neutral red, MTT and protein assay in hepatoma cell lines following exposure to cadmium chloride. *Toxicol. Lett.* 160 171–177. 10.1016/j.toxlet.2005.07.001 16111842

[B20] Genilloud O. (2017). Actinomycetes: still a source of novel antibiotics. *Nat. Prod. Rep.* 34 1203–1232. 10.1039/c7np00026j 28820533

[B21] Guerrero-Garzón J. F. Zehl M. Schneider O. Rückert C. Busche T. Kalinowski (2020). *Streptomyces* spp. from the marine sponge Antho dichotoma: analyses of secondary metabolite biosynthesis gene clusters and some of their products. *Front. Microbiol.* 11:437. 10.3389/fmicb.2020.00437 32256483 PMC7093587

[B22] Hamedi J. Mohammadipanah F. Ventosa A. (2013). Systematic and biotechnological aspects of halophilic and halotolerant actinomycetes. *Extremophiles* 17 1–13. 10.1007/s00792-012-0493-5 23129307

[B23] Harvey A. L. Edrada-Ebel R. Quinn R. J. (2015). The re-emergence of natural products for drug discovery in the genomics era. *Nat. Rev. Drug Discov.* 14 111–129. 10.1038/nrd4510 25614221

[B24] Hertweck C. Luzhetskyy A. Rebets Y. Bechthold A. (2007). Type II polyketide synthases: gaining a deeper insight into enzymatic teamwork. *Nat. Prod. Rep.* 24 162–190. 10.1039/b507395m 17268612

[B25] Jensen P. R. Mincer T. J. Williams P. G. Fenical W. (2005). Marine actinomycete diversity and natural product discovery. *Antonie Van Leeuwenhoek* 87 43–48. 10.1007/s10482-004-6540-1 15726290

[B26] Katz M. Hover B. M. Brady S. F. (2016). Culture-independent discovery of natural products from soil metagenomes. *J. Ind. Microbiol. Biotechnol.* 43 129–141. 10.1007/s10295-015-1706-6 26586404

[B27] Kim M. C. Machado H. Jang K. H. Trzoss L. Jensen P. R. Fenical W. (2018). Integration of genomic data with NMR analysis enables assignment of the full stereostructure of neaumycin B, a potent inhibitor of glioblastoma from a marine-derived Micromonospora. *J. Am. Chem. Soc.* 140 10775–10784. 10.1021/jacs.8b04848 30085661 PMC6533909

[B28] Koskiniemi H. Metsä-Ketelä M. Dobritzsch D. Kallio P. Korhonen H. Mäntsälä P. (2007). Crystal structures of two aromatic hydroxylases involved in the early tailoring steps of angucycline biosynthesis. *J. Mol. Biol.* 372 633–648. 10.1016/j.jmb.2007.06.087 17669423

[B29] Kurapova A. I. Zenova G. M. Sudnitsyn I. I. Kizilova A. K. Manucharova N. A. Norovsuren Z. (2012). Thermotolerant and thermophilic actinomycetes from soils of Mongolia desert steppe zone. *Microbiology* 81 98–108. 10.1134/S0026261712010092 22629687

[B30] Kutzner H. J. Waksman S. A. (1959). *Streptomyces coelicolor* Müller and *Streptomyces violaceoruber* Waksman and Curtis, two distinctly different organisms. *J. Bacteriol.* 78 528–538. 10.1128/jb.78.4.528-538.1959 14412998 PMC290581

[B31] Lee J. Han C. Lee T. G. Chin J. Choi H. Lee W. (2016). Marinopyrones A-D, α-pyrones from marine-derived actinomycetes of the family Nocardiopsaceae. *Tetrahedron Lett.* 57 1997–2000. 10.1016/j.tetlet.2016.03.084

[B32] Lee N. Kim W. Hwang S. Lee Y. Cho S. Palsson B. (2020). Thirty complete *Streptomyces* genome sequences for mining novel secondary metabolite biosynthetic gene clusters. *Sci. Data.* 7:55. 10.1038/s41597-020-0395-9 32054853 PMC7018776

[B33] Mahajan G. B. Balachandran L. (2012). Antibacterial agents from actinomycetes - A review. *Front. Biosci.* 4E 240–253. 10.2741/e373 22201868

[B34] Malmierca M. G. González-Montes L. Pérez-Victoria I. Sialer C. Braña A. F. García Salcedo R. (2018). Searching for glycosylated natural products in actinomycetes and identification of novel macrolactams and angucyclines. *Front. Microbiol.* 9:39. 10.3389/fmicb.2018.00039 29441046 PMC5797532

[B35] Martin P. Rodier S. Mondon M. Renoux B. Pfeiffer B. Renard P. (2002). Synthesis and cytotoxic activity of tetracenomycin D and of saintopin analogues. *Bioorg. Med. Chem.* 10 253–260. 10.1016/S0968-0896(01)00273-5 11741773

[B36] McDaniel R. Khosla C. Hutchinson C. R. (1995). Engineered biosynthesis of novel polyketides: analysis of TcmN function in tetracenomycin biosynthesis. *J. Am. Chem. Soc.* 117 6805–6810. 10.1021/ja00131a001

[B37] Medema M. H. Kottmann R. Yilmaz P. Cummings M. Biggins J. B. Blin K. (2015). Minimum information about a biosynthetic gene cluster. *Nat. Chem. Biol.* 11 625–631. 10.1038/nchembio.1890 26284661 PMC5714517

[B38] Medema M. H. Takano E. Breitling R. (2013). Detecting sequence homology at the gene cluster level with multigeneblast. *Mol. Biol. Evol.* 30 1218–1223. 10.1093/molbev/mst025 23412913 PMC3670737

[B39] Metelev M. Tietz J. I. Melby J. O. Blair P. M. Zhu L. Livnat I. (2015). Structure, bioactivity, and resistance mechanism of streptomonomicin, an unusual lasso peptide from an understudied halophilic actinomycete. *Chem. Biol.* 22 241–250. 10.1016/j.chembiol.2014.11.017 25601074 PMC4336579

[B40] Metsä-Ketelä M. Halo L. Munukka E. Hakala J. Mäntsäla P. Ylihonko K. (2002). Molecular evolution of aromatic polyketides and comparative sequence analysis of polyketide ketosynthase and 16S ribosomal DNA genes from various *Streptomyces* species. *Appl. Environ. Microbiol.* 68 4472–4479. 10.1128/AEM.68.9.4472-4479.2002 12200302 PMC124067

[B41] Mincer T. J. Jensen P. R. Kauffman C. A. Fenical W. (2002). Widespread and persistent populations of a major new marine actinomycete taxon in ocean sediments. *Appl. Environ. Microbiol.* 68 5005–5011. 10.1128/AEM.68.10.5005-5011.2002 12324350 PMC126404

[B42] Mohammadipanah F. Wink J. (2016). Actinobacteria from arid and desert habitats: diversity and biological activity. *Front. Microbiol.* 6:1541. 10.3389/fmicb.2015.01541 26858692 PMC4729944

[B43] Monciardini P. (2002). New PCR primers for the selective amplification of 16S rDNA from different groups of actinomycetes. *FEMS Microbiol. Ecol.* 42 419–429. 10.1016/S0168-6496(02)00353-7 19709301

[B44] Montalvo N. F. Mohamed N. M. Enticknap J. J. Hill R. T. (2005). Novel actinobacteria from marine sponges. *Antonie Van Leeuwenhoek* 87 29–36. 10.1007/s10482-004-6536-x 15726288

[B45] Najafi A. Moradinasab M. Nabipour I. (2018). First record of microbiomes of sponges collected from the Persian Gulf, using tag pyrosequencing. *Front. Microbiol.* 9:1500. 10.3389/fmicb.2018.01500 30034382 PMC6043863

[B46] Naughton L. M. Romano S. O’Gara F. Dobson A. D. W. (2017). Identification of secondary metabolite gene clusters in the Pseudovibrio genus reveals encouraging biosynthetic potential toward the production of novel bioactive compounds. *Front. Microbiol.* 8:1494. 10.3389/fmicb.2017.01494 28868049 PMC5563371

[B47] Newman D. J. Cragg G. M. (2016). Natural products as sources of new drugs from 1981 to 2014. *J. Nat. Prod.* 79 629–661. 10.1021/acs.jnatprod.5b01055 26852623

[B48] Nikodinovic J. Barrow K. D. Chuck J. A. (2003). High yield preparation of genomic DNA from *Streptomyces*. *Biotechniques* 35 932–936. 10.2144/03355bm05 14628665

[B49] Passari A. K. Leo V. V. Chandra P. Kumar B. Nayak C. Hashem A. (2018). Bioprospection of actinobacteria derived from freshwater sediments for their potential to produce antimicrobial compounds. *Microb. Cell Fact.* 17 1–14. 10.1186/s12934-018-0912-0 29729667 PMC5935920

[B50] Pfefferle C. Breinholt J. Gürtler H. Fiedler H.-P. (1997). 1-Hydroxy-4-methoxy-2-naphthoic acid, a herbicidal compound produced by Streptosporangium cinnabarinum ATCC 31213. *J. Antibiot.* 50 1067–1068. 10.7164/antibiotics.50.1067 9510917

[B51] Pye C. R. Bertin M. J. Lokey R. S. Gerwick W. H. Linington R. G. (2017). Retrospective analysis of natural products provides insights for future discovery trends. *Proc. Natl. Acad. Sci. U.S.A.* 114 5601–5606. 10.1073/pnas.1614680114 28461474 PMC5465889

[B52] Qu X. Y. Ren J. W. Peng A. H. Lin S. Q. Lu D. D. Du Q. Q. (2019). Cytotoxic, anti-migration, and anti-invasion activities on breast cancer cells of angucycline glycosides isolated from a marine-derived *Streptomyces* sp. *Mar. Drugs* 17:E277. 10.3390/md17050277 31075906 PMC6562490

[B53] Raimundo I. Silva S. Costa R. Keller-Costa T. (2018). Bioactive secondary metabolites from octocoral-associated microbes—new chances for blue growth. *Mar. Drugs* 16:E485. 10.3390/md16120485 30518125 PMC6316421

[B54] Ramos A. Lombo F. Brana A. F. Rohr J. Mendez C. Salas J. A. (2008). Biosynthesis of elloramycin in *Streptomyces olivaceus* requires glycosylation by enzymes encoded outside the aglycon cluster. *Microbiology* 154 781–788. 10.1099/mic.0.2007/014035-0 18310024

[B55] Riegl B. M. Purkis S. J. (2012). “Coral reefs of the Gulf: adaptation to climatic extremes in the world’s hottest sea,” in *Coral Reefs of the Gulf. Coral Reefs of the World*, eds Riegl B. Purkis S. (Cham: Springer), 1–4. 10.1007/978-94-007-3008-3_1

[B56] Rohr J. Eick S. Zeeck A. Reuschenbach P. Zahner H. Fiedler H. P. (1988). Metabolic products of microorganisms. 249. Tetracenomycins B3 and D3, key intermediates of the elloramycin and tetracenomycin C biosynthesis. *J. Antibiot.* 41 1066–1073. 10.7164/antibiotics.41.1066 3170342

[B57] Schorn M. A. Alanjary M. M. Aguinaldo K. Korobeynikov A. Podell S. Patin N. (2016). Sequencing rare marine actinomycete genomes reveals high density of unique natural product biosynthetic gene clusters. *Microbiology* 162 2075–2086. 10.1099/mic 27902408 PMC5756490

[B58] Sciara G. Kendrew S. G. Miele A. E. Marsh N. G. Federici L. Malatesta F. (2003). The structure of ActVA-Orf6, a novel type of monooxygenase involved in actinorhodin biosynthesis. *EMBO J.* 22 205–215. 10.1093/emboj/cdg031 12514126 PMC140106

[B59] Seipke R. F. Kaltenpoth M. Hutchings M. I. (2012). *Streptomyces* as symbionts: an emerging and widespread theme? *FEMS Microbiol. Rev.* 36 862–876. 10.1111/j.1574-6976.2011.00313.x 22091965

[B60] Sekurova O. N. Schneider O. Zotchev S. B. (2019). Novel bioactive natural products from bacteria via bioprospecting, genome mining and metabolic engineering. *Microb. Biotechnol.* 12 828–844. 10.1111/1751-7915.13398 30834674 PMC6680616

[B61] Shen Y. Yoon P. Yu T. W. Floss H. G. Hopwood D. Moore B. S. (1999). Ectopic expression of the minimal whiE polyketide synthase generates a library of aromatic polyketides of diverse sizes and shapes. *Proc. Natl. Acad. Sci. U.S.A.* 96 3622–3627. 10.1073/pnas.96.7.3622 10097087 PMC22344

[B62] Siezen R. J. Khayatt B. I. (2008). Natural products genomics. *Microb. Biotechnol.* 1 275–282. 10.1111/j.1751-7915.2008.00044.x 21261848 PMC3815393

[B63] Simão F. A. Waterhouse R. M. Ioannidis P. Kriventseva E. V. Zdobnov E. M. (2015). BUSCO: assessing genome assembly and annotation completeness with single-copy orthologs. *Bioinformatics* 31 3210–3212. 10.1093/bioinformatics/btv351 26059717

[B64] Subramani R. Aalbersberg W. (2013). Culturable rare actinomycetes: diversity, isolation and marine natural product discovery. *Appl. Microbiol. Biotechnol.* 97 9291–9321. 10.1007/s00253-013-5229-7 24057404

[B65] Sun W. Zhang F. He L. Karthik L. Li Z. (2015). Actinomycetes from the South China Sea sponges: isolation, diversity, and potential for aromatic polyketides discovery. *Front. Microbiol.* 6:1048. 10.3389/fmicb.2015.01048 26483773 PMC4589764

[B66] Tamura K. Stecher G. Peterson D. Filipski A. Kumar S. (2013). MEGA6: molecular evolutionary genetics analysis version 6.0. *Mol. Biol. Evol.* 30 2725–2729. 10.1093/molbev/mst197 24132122 PMC3840312

[B67] Taylor M. W. Radax R. Steger D. Wagner M. (2007). Sponge-associated microorganisms: evolution, ecology, and biotechnological potential. *Microbiol. Mol. Biol. Rev.* 71 295–347. 10.1128/mmbr.00040-06 17554047 PMC1899876

[B68] Thomas T. R. A. Kavlekar D. P. LokaBharathi P. A. (2010). Marine drugs from sponge-microbe association—A review. *Mar. Drugs* 8 1417–1468. 10.3390/md8041417 20479984 PMC2866492

[B69] Thompson T. B. Katayama K. Watanabe K. Hutchinson C. R. Rayment I. (2004). Structural and functional analysis of tetracenomycin F2 cyclase from *Streptomyces glaucescens*: a type II polyketide cyclase. *J. Biol. Chem.* 279 37956–37963. 10.1074/jbc.M406144200 15231835

[B70] Tsai I. J. Otto T. D. Berriman M. (2010). Improving draft assemblies by iterative mapping and assembly of short reads to eliminate gaps. *Genome Biol.* 11:R41. 10.1186/gb-2010-11-4-r41 20388197 PMC2884544

[B71] Vaughan G. O. Burt J. A. (2016). The changing dynamics of coral reef science in Arabia. *Mar. Pollut. Bull.* 105 441–458. 10.1016/j.marpolbul.2015.10.052 26621575

[B72] Wu C. Kim H. K. van Wezel G. P. Choi Y. H. (2015). Metabolomics in the natural products field – a gateway to novel antibiotics. *Drug Discov. Today Technol.* 13 11–17. 10.1016/j.ddtec.2015.01.004 26190678

[B73] Xu D. Han L. Li C. Cao Q. Zhu D. Barrett N. H. (2018). Bioprospecting deep-sea actinobacteria for novel anti-infective natural products. *Front. Microbiol.* 9:787. 10.3389/fmicb.2018.00787 29760684 PMC5936781

[B74] Xu X. N. Chen L. Y. Chen C. Tang Y. J. Bai F. W. (2018). Genome mining of the marine actinomycete *Streptomyces* sp. DUT11 and discovery of tunicamycins as anti-complement agents. *Front. Microbiol.* 9:1318. 10.3389/fmicb.2018.01318 29973921 PMC6019454

[B75] Yue S. Motamedi H. Wendt-Pienkowski E. Hutchinson C. R. (1986). Anthracycline metabolites of Tetracenomycin C-nonproducing *Streptomyces glaucescens* mutants. *J. Bact.* 167 581–586. 10.1128/jb.167.2.581-586.1986 3460987 PMC212929

[B76] Zhang D. F. Pan H. Q. He J. Zhang X. M. Zhang Y. G. Klenk H. P. (2013). Description of *Streptomonospora sediminis* sp. nov. and *Streptomonospora nanhaiensis* sp. nov., and reclassification of *Nocardiopsis arabia* Hozzein & Goodfellow 2008 as *Streptomonospora arabica* comb. nov. and emended description. *Int. J. Syst. Evol. Microbiol.* 63 4447–4455. 10.1099/ijs.0.052704-0 23847283

[B77] Zhang G. Zhang Y. Yin X. Wang S. (2015). *Nesterenkonia alkaliphila* sp. nov., an alkaliphilic, halotolerant actinobacteria isolated from the western Pacific Ocean. *Int. J. Syst. Evol. Microbiol.* 65 516–521. 10.1099/ijs.0.065623-0 25389152

[B78] Zou H. Chen N. Shi M. Xian M. Song Y. Liu J. (2016). The metabolism and biotechnological application of betaine in microorganism. *Appl. Microbiol. Biotechnol.* 100 3865–3876. 10.1007/s00253-016-7462-3 27005411

