# Supplementary material for: Genotyping-Guided Discovery of Persiamycin A From Sponge-Associated Halophilic Streptomonospora sp. PA3
Source: Front Microbiol. 2020 Jun 9;11:1237. doi: 10.3389/fmicb.2020.01237 (PMC7296137; doi:10.3389/fmicb.2020.01237)
Supplement: Supplementary file 1 [file Presentation_1.pdf]

Supplementary Material

Table of contents

Table S1. NMR data for 1-hydroxy-4-methoxy-2-naphthoic acid (**1**). 2
Figure S1. Main HMBC correlations of **1**. 2
Table S2. NMR data for Persiamycin A (**2**). 3
Figure S2. Main HMBC correlations of **2**. 3
Figure S3. Proton spectrum of **1** in MeOD. 4
Figure S4. Carbon spectrum of **1** in DMSO-*d6.* 4
Figure S5. COSY spectrum of **1** in DMSO-*d6*. 5
Figure S6. HSQCDE spectrum of **1** in MeOD. 5
Figure S7. HMBC spectrum of **1** in DMSO-*d6*. 6
Figure S8. Proton spectrum of **2** in DMSO-*d6* and $CDCl_3$. 6
Figure S9. Carbon spectrum of **2** in DMSO-*d6* and $CDCl_3$. 7
Figure S10. HSQCDE spectrum of **2** in DMSO-*d6* and $CDCl_3$. 7
Figure S11. HMBC spectrum of **2** in DMSO-*d6* and $CDCl_3$. 8

Table S1. NMR data for 1-hydroxy-4-methoxy-2-naphthoic acid (**1**). ^{1}H and ^{13}C chemical shifts and HMBC correlations for 1-hydroxy-4-methoxy-2-naphthoic acid. Chemical shifts are internally referenced to TMS. ^{1}H Spectrum recorded in MeOD (600 MHz) and ^{13}C spectrum (150 MHz) in DMSO-*d6*.

Position	δ ppm ^{13}C	δ ppm, *J* Hz ^{1}H	HMBC
1-OH	155.2	Exchange	
2	109.6		
3	103.8	7.17 s	C1, C2, C4, C4a, C5
4	144.9		
4a	127.9		
5	121.2	8.12 ddd, 0.8, 1.2, 8.3	C4, C7, C8a
6	127.1	7.53 ddd, 1.2, 7.1, 8.3	C4a, C5, C8, C8a
7	125.1	7.48 ddd, 1.2, 7.1, 8.3	C4a, C5, C8, C8a
8	123.2	8.27 ddd, 0.8, 1.2, 8.3	C1, C4a, C6
8a	125.8		
9-COOH	172.5	Exchange	
10	55.4	3.96 s, 3H	C3, C4, C10

s=singlet, d=doublet, integrals are 1H unless otherwise stated.

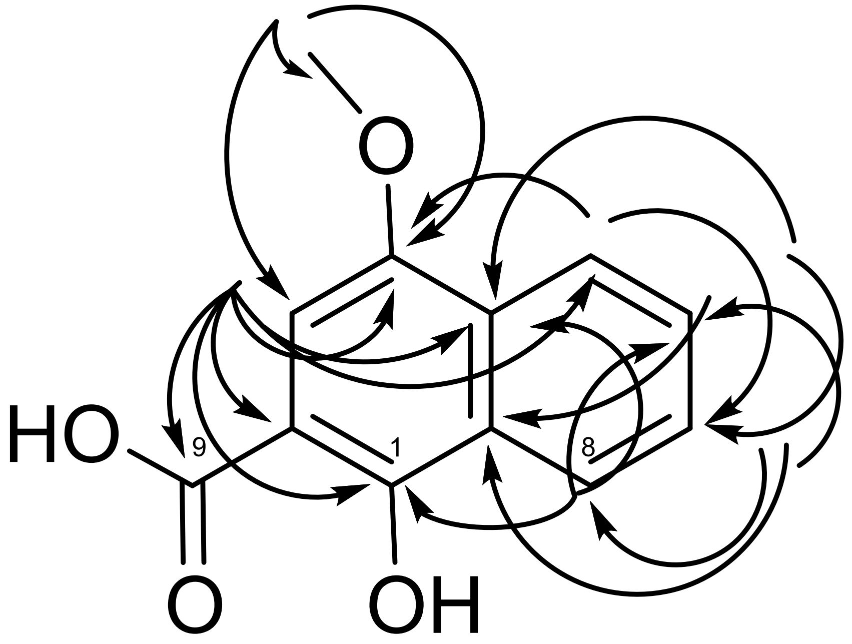

Figure S1. Main HMBC correlations of **1**.

Table S2. NMR data for Persiamycin A (**2**). ^{1}H (600 MHz) and ^{13}C (125 MHz) chemical shifts and HMBC correlations for Persiamycin A. Chemical shifts are internally referenced to TMS. Spectra are recorded in a mix of DMSO-*d6* and $CDCl_3$.

	δ ppm	δ ppm, *J* Hz	HMBC
Position	^{13}C	^{1}H	
1-OH	158.0	14.7*	
2	124.6		
2-CH_3	9.2	2.27 br, 3H	C1, C2, C3, C4, C2-CH_3
3-OH	161.8	10.22*	
4	112.2	8.08 br	C2, C5, C12a
4a	132.2		
5	181.2		
5a	128.3		
6	121.6	7.95 br	C5, C7, C10a, C11a
6a	140.4		
7	111.6	7.13 br	C6, C9, C10a
8-OH	167.2	10.22*	
9	123.3	6.98 br	C7, C10a, C10-CH_3
10	142.1		
10-CH_3	24.8	2.93 br, 3H	C9, C10, C10a, C10-CH_3
10a	120.0		
11-OH	159.9	12.80*	
11a	107.2		
12	189.7		
12a	112.1		

*partial exchange, br=broad, integrals are 1H unless otherwise stated.

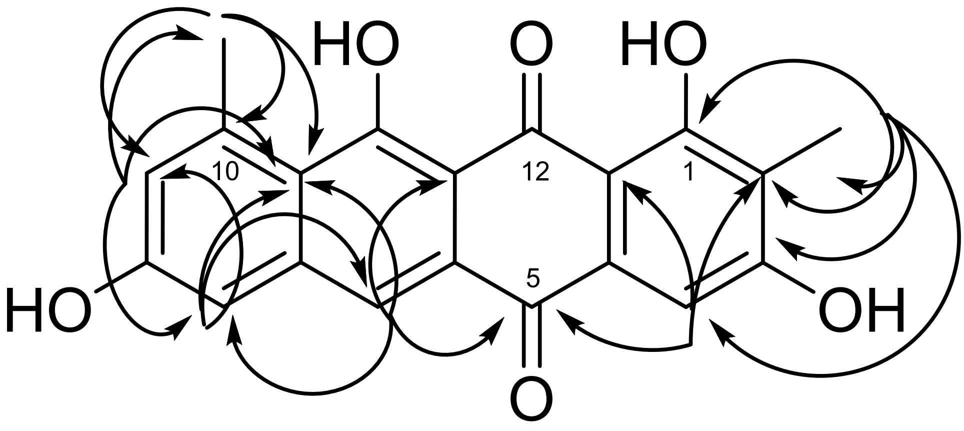

Figure S2. Main HMBC correlations of **2**.

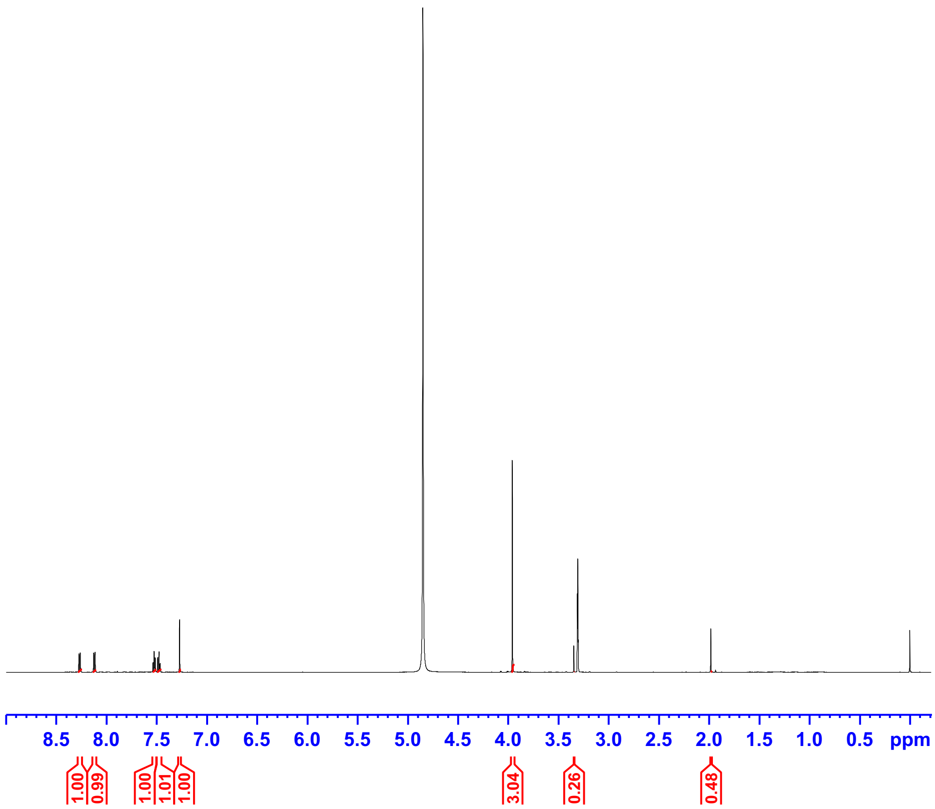

Figure S3. Proton spectrum of **1** in MeOD.

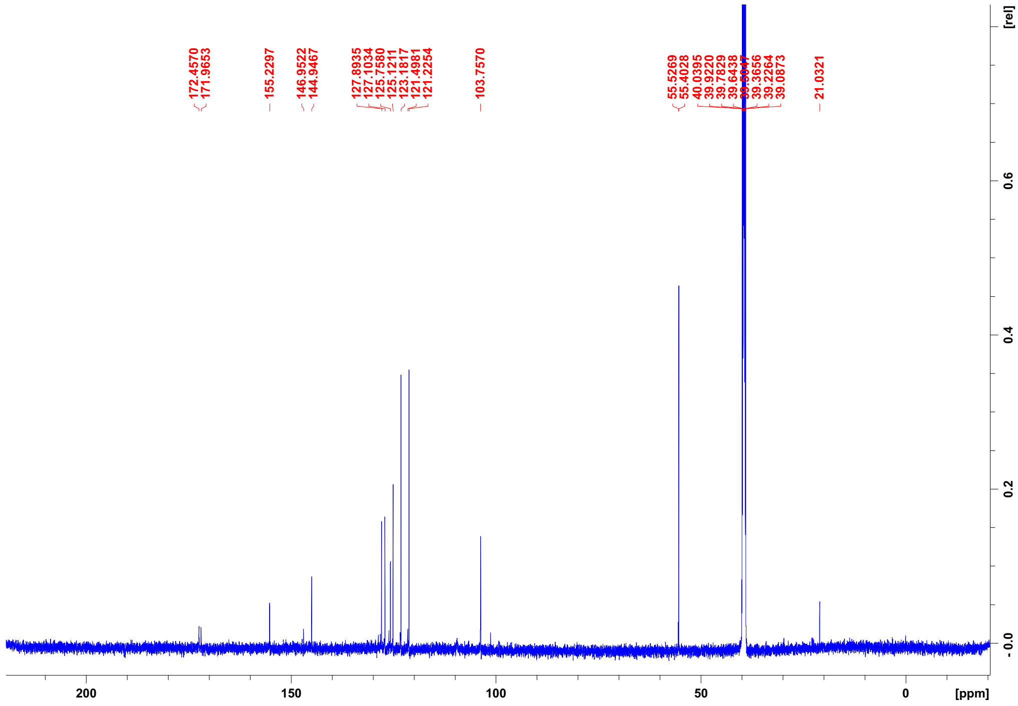

Figure S4. Carbon spectrum of **1** in DMSO-*d6*.

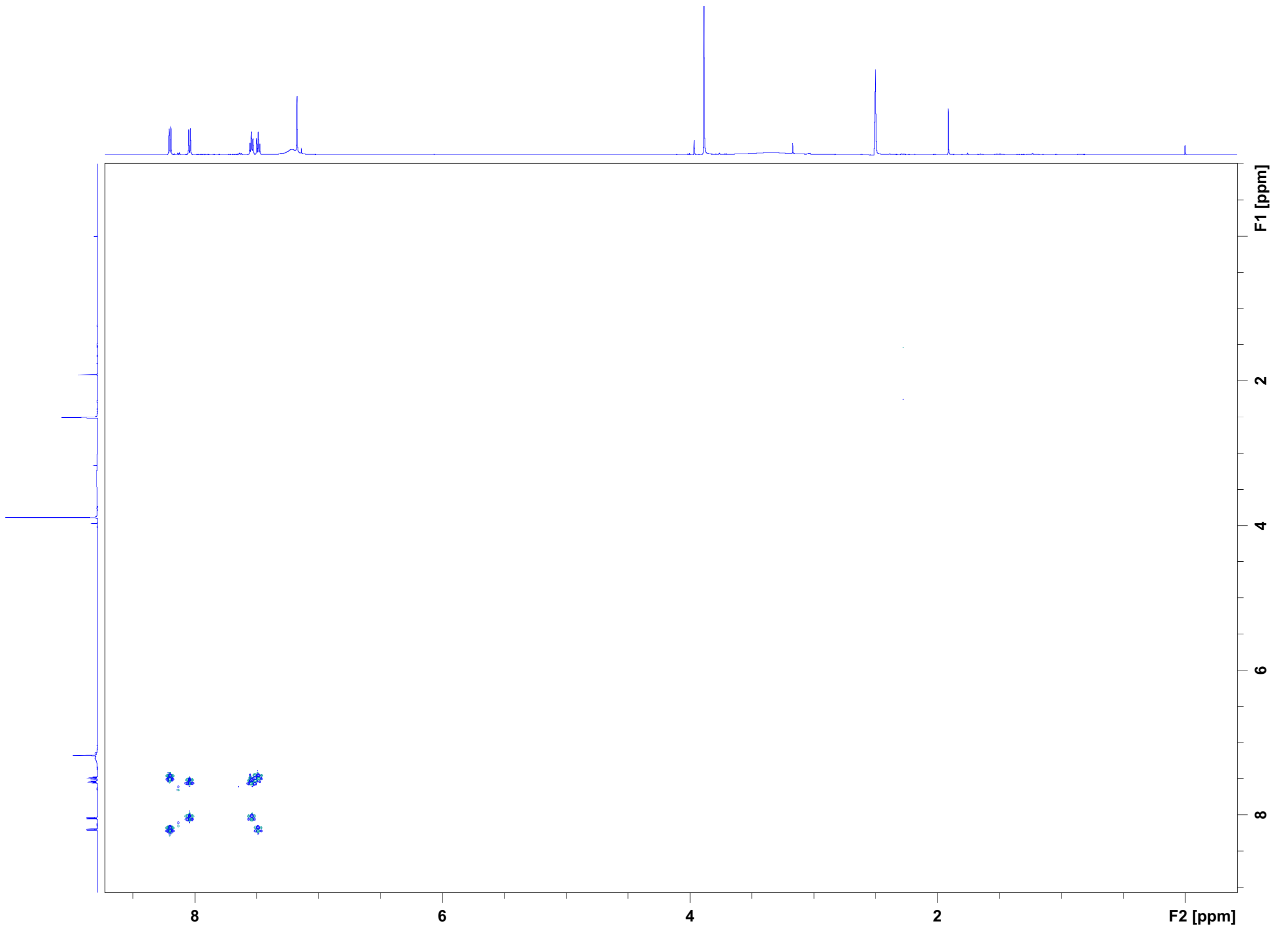

Figure S5. COSY spectrum of **1** in DMSO-*d6*.

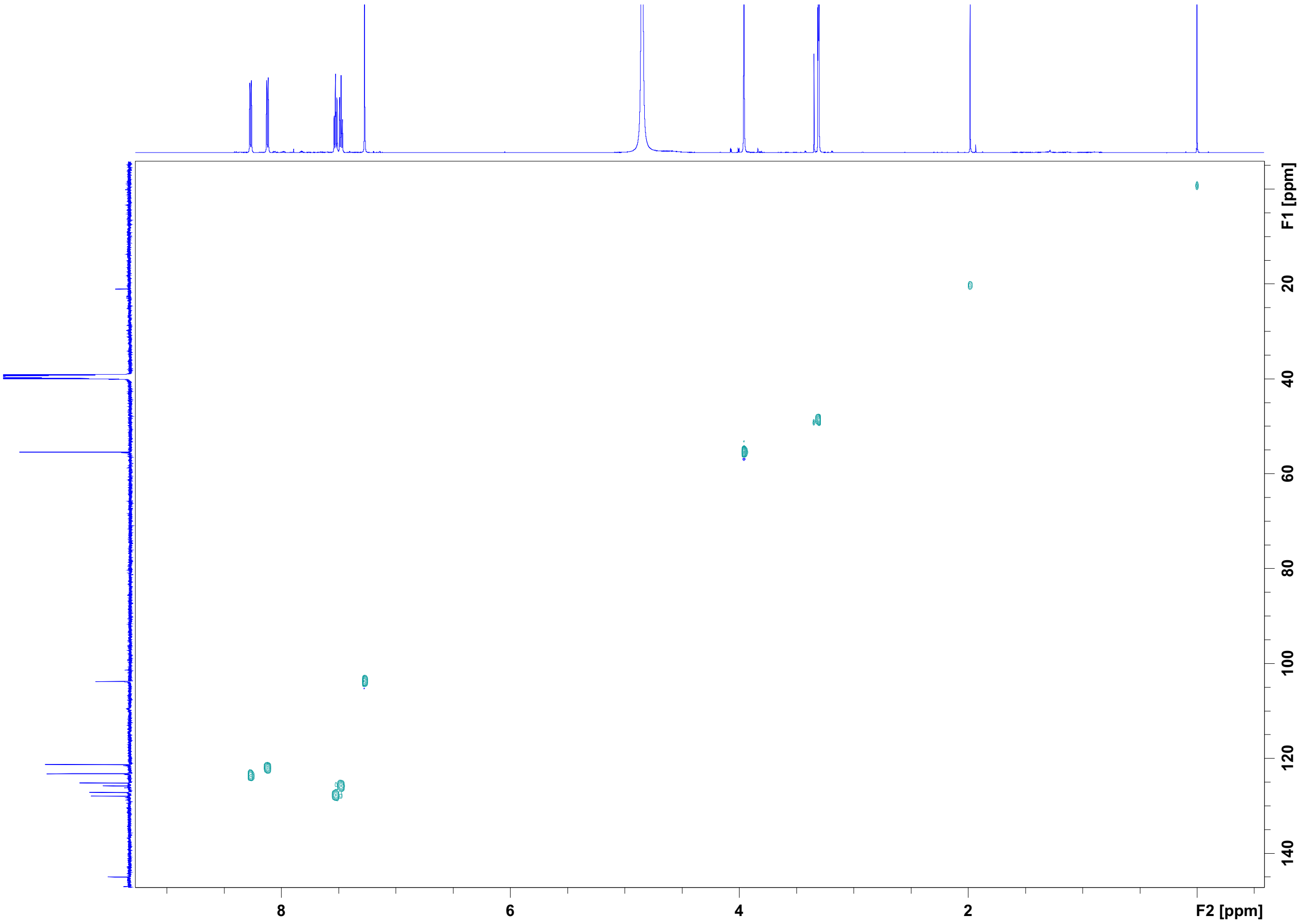

Figure S6. HSQCDE spectrum of **1** in MeOD.

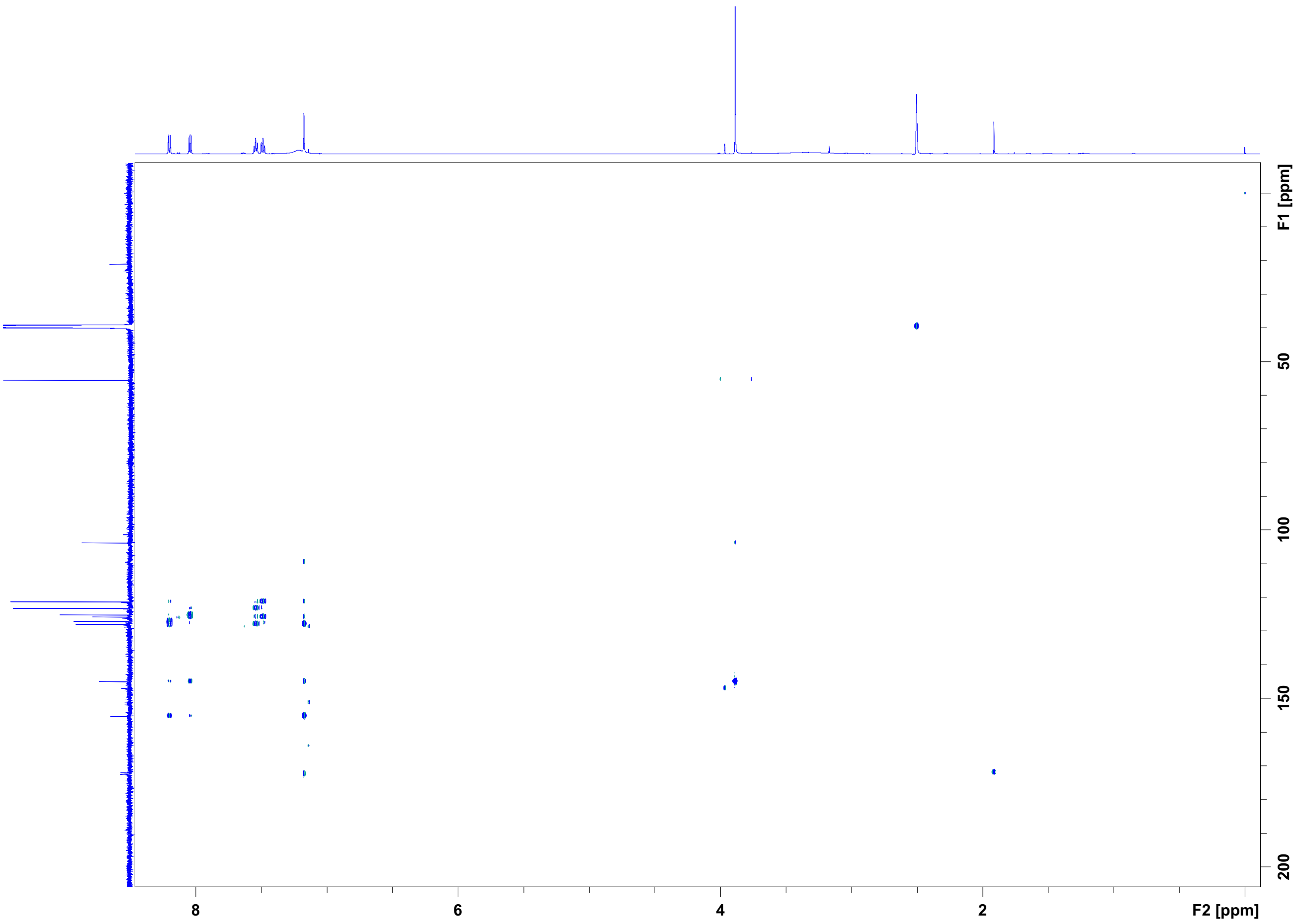

Figure S7. HMBC spectrum of **1** in DMSO-*d6*.

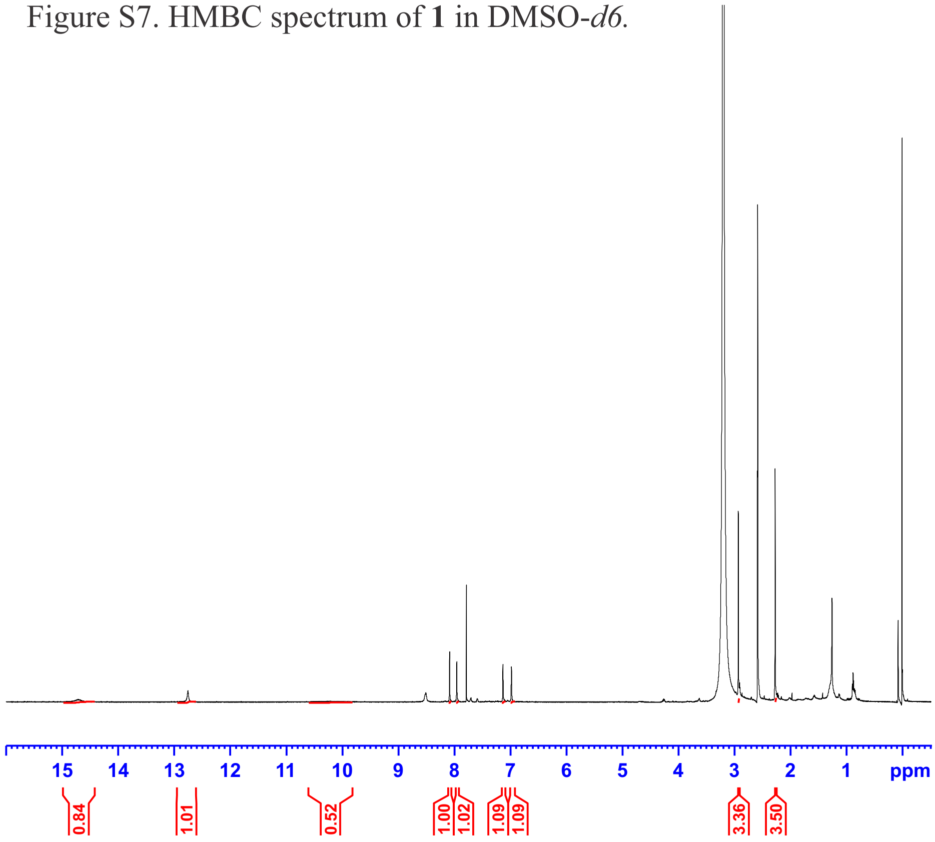

Figure S8. Proton spectrum of **2** in DMSO-*d6* and $CDCl_3$.

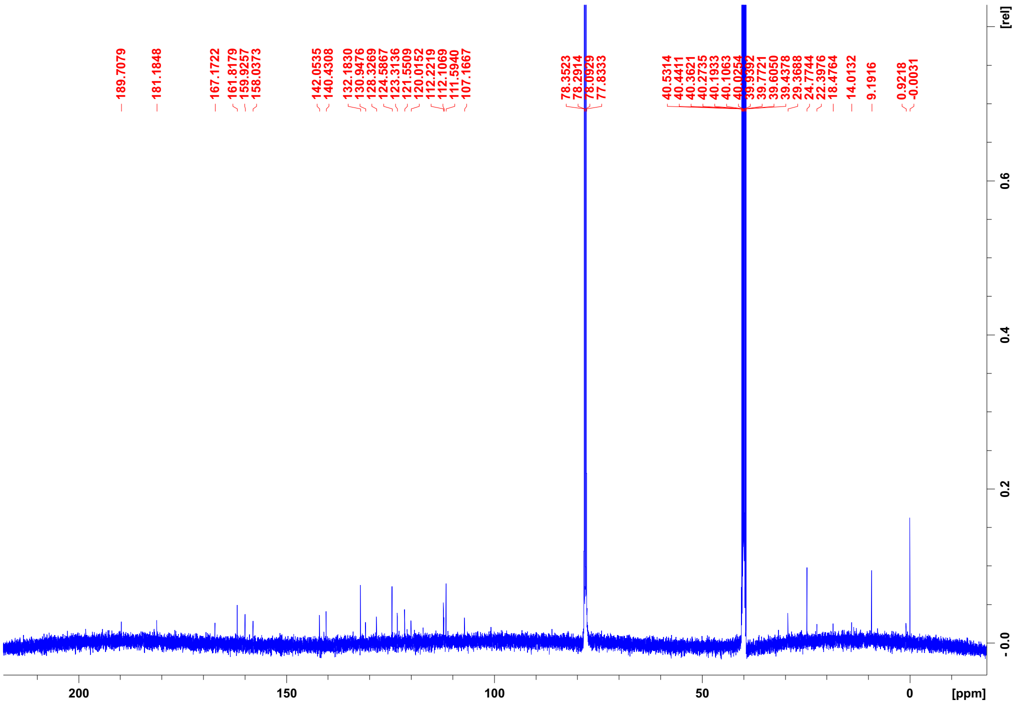

Figure S9. Carbon spectrum of **2** in DMSO-*d6* and $CDCl_3$.

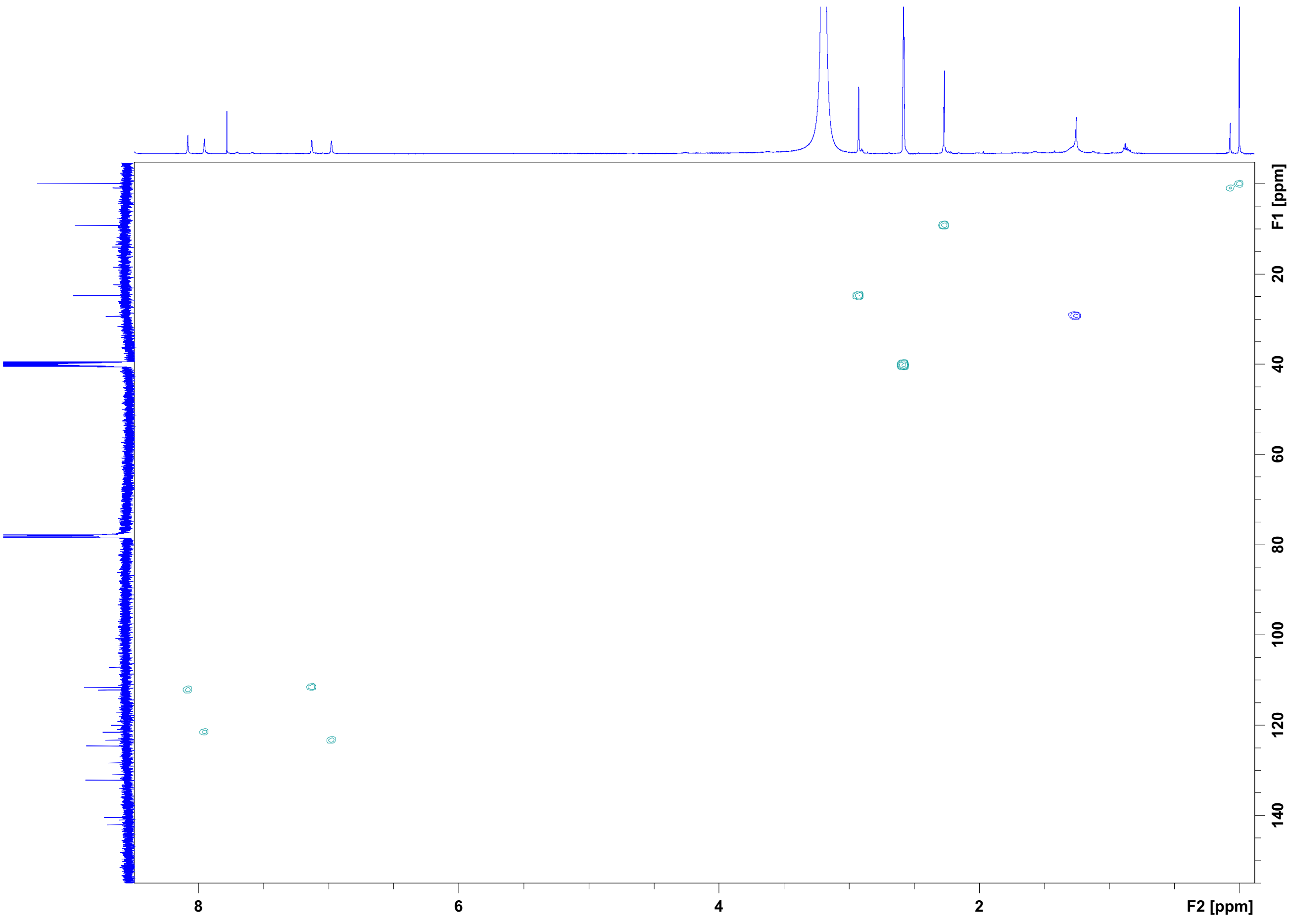

Figure S10. HSQCDE spectrum of **2** in DMSO-*d6* and $CDCl_3$.

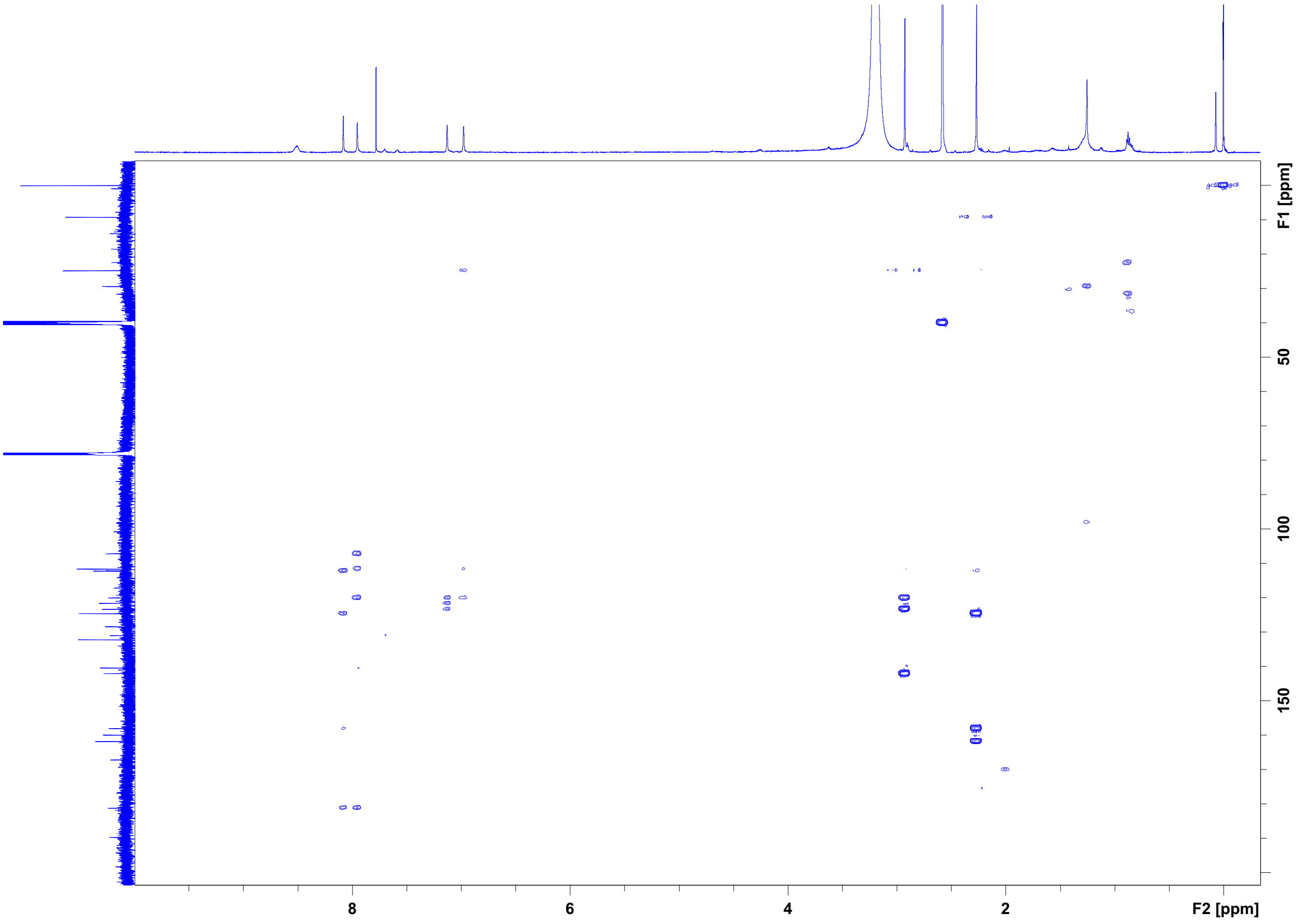

Figure S11. HMBC spectrum of **2** in DMSO-*d6* and $CDCl_3$.
